# Feasibility of preoperative supervised home-based exercise in older adults undergoing colorectal cancer surgery – A randomized controlled design

**DOI:** 10.1371/journal.pone.0219158

**Published:** 2019-07-02

**Authors:** Emelie Karlsson, Parastou Farahnak, Erika Franzén, Malin Nygren-Bonnier, Jaap Dronkers, Nico van Meeteren, Elisabeth Rydwik

**Affiliations:** 1 Karolinska Institutet, Department of Neurobiology, Care Sciences and Society, Division of Physiotherapy, Huddinge, Sweden; 2 Department of Clinical Science and Education, and Department of Colorectal Surgery, Södersjukhuset, Stockholm, Sweden; 3 Allied Health Professionals, Function Area Occupational Therapy & Physiotherapy, Karolinska University Hospital, Stockholm, Sweden; 4 Stockholms Sjukhem R&D Unit, Stockholm, Sweden; 5 Research group Innovation of Movement Care, University of Applied Sciences, Utrecht, the Netherlands; 6 Department of Physical Therapy, Gelderse Vallei Hospital, Ede, the Netherlands; 7 Top Sector Life Sciences & Health (Health~Holland), the Hague, the Netherlands; 8 Department of Epidemiology and Research School CAPHRI, Maastricht University, Maastricht, the Netherlands; 9 Stockholm County Council, FOU nu, Research and Development unit for the elderly, Järfälla, Sweden; German Sport University Cologne, GERMANY

## Abstract

Preoperative physical exercise is emerging as a growing field of research globally. There are still challenges in recruiting vulnerable older people, and time constraints in preoperative cancer care to consider. We therefore evaluated the feasibility of short-term supervised home-based exercise in older people prior to colorectal cancer surgery. This feasibility study was conducted between September 2016 and June 2018. People ≥70 years scheduled for colorectal cancer surgery were recruited and randomized to an intervention group receiving supervised home-based physical exercise at a high level of estimated exertion or a standard care group following the standard preoperative path. The exercise (respiratory, strength, and aerobic) consisted of 2–3 supervised sessions a week in the participants’ homes, for at least 2–3 weeks or until surgery, and a self-administered exercise program in between. The primary outcome was process feasibility, including aspects specifically related to recruitment rate, compliance to the intervention, and acceptability. The secondary outcome was scientific feasibility including treatment safety, description of dose level and response, and estimation of treatment results. Twenty-three participants were included (recruitment rate 35%). A median of 6 supervised sessions was conducted over a 17-day exercise period. Compliance with the supervised sessions was 97%, and participants found the intervention acceptable. Concerning the self-administered exercise, a median of 19 inspiratory muscle training, 6 functional strength, and 8.5 aerobic sessions were reported. Challenges reported by program instructors were time constraints and difficulties in achieving high exercise intensities on the Borg CR-10 scale. A statistically significant between-group difference was only found in inspiratory muscle strength, favoring the intervention group (p<0.01). A short-term preoperative supervised home-based physical exercise intervention can be conducted, with respect to compliance and acceptability, in older people with similar physical status as in this study prior to colorectal cancer surgery. However, modifications are warranted with respect to improving recruitment rates and achieving planned intensity levels prior to conducting a definitive trial.

## Introduction

Colorectal cancer is the third most common form of cancer worldwide, with its incidence increasing with age [1]. The primary treatment for colorectal cancer is surgical interventions where older people receive surgery to a greater extent today than in the past [2]. The disease, hospitalization, and surgical stress are all factors which could result in reduced cardiopulmonary capacity and muscle function [3]. Older people planned for surgery may have different needs and outcomes compared to their younger counterparts due to differences in physiology, limited reserve capacity, heterogeneity, and a higher presence of comorbidities with increasing chronological age. All this may lead to an increased risk of complications, functional decline, and decreased health-related quality of life [4]. Therefore, it is crucial for this patient group to be prepared preoperatively to optimize health and function.

The concept of prehabilitation, in which preoperative physical fitness plays an essential role, is an emerging field of research globally. Within cancer research, prehabilitation is defined as “a process on the cancer continuum of care that occurs between the time of cancer diagnosis and the beginning of acute treatment”, emphasizing the time sensitive component [5]. For people undergoing abdominal cancer surgery, preoperative exercise such as aerobic capacity and inspiratory muscle training (IMT) seems feasible, improves physical capacity, and has shown the potential to reduce postoperative complications [6–13]. However, reviews imply that further research is needed to identify and evaluate the optimal intervention regarding type, as well as duration and intensity of exercise [6, 7, 14, 15]. To this extent, the Consensus on Therapeutic Exercise Training (CONTENT) scale is recommended to assess therapeutic validity and to organize the most prominent exercise components [16]. A recent study was the first to focus on prehabilitation in high-risk patients [8]. Nevertheless, the study did not solely involve cancer surgery, and the intervention time was 6 weeks, which is longer than the current preoperative time constraints in Sweden and many other countries where time from surgery decision to the actual surgery taking place is decreasing. To this end, high-intensity preoperative training has been suggested due to time constraints [10, 17].

There are still challenges in recruiting vulnerable older people as eligible patients often decline participation [18]. Also, compliance with preoperative exercise programs might be poor, which likely inhibits a clear view of the program’s effects on outcomes [6, 7]. Home-based supervised exercise programs could be one way of increasing adherence rates among older individuals [19–21], and to meet frequently mentioned objections concerning transportation [22]. In addition, interventions involving older people should focus on improving outcomes of importance to the older person, which means functional training, defined as “training that has a meaning to maintain or improve performance of a specific goal or task in daily life” [23]. Therefore, we have designed an intervention with a home-based, functional exercise program in the participant’s habitual daily environment. The aim of this study was, prior to a larger trial, to evaluate the feasibility of a preoperative, supervised home-based physical exercise program at a high level of estimated exertion, in older people undergoing colorectal cancer surgery in Sweden: “Can it be done? Should it be done? And if so, how?” [24–26]

## Materials and methods

### Participants and study design

We conducted a randomized feasibility study in preparation for a planned two-arm randomized controlled trial with a 1:1 allocation ratio. Patients scheduled for colorectal cancer surgery at Stockholm South General Hospital were informed about the study and asked to participate. The screening for inclusion was undertaken from September 2016 to June 2018 by a surgeon and nurse at the weekly colorectal cancer conferences (CRC). There were two interruptions in recruitment (totaling 3 months) due to limited staff compliment during the Christmas and summer vacation periods, respectively.

The inclusion criteria were: (1) Age ≥70 years; (2) ability to understand and speak the Swedish language; (3) scheduled for surgery due to colorectal cancer or suspected colorectal cancer. The exclusion criteria were: (1) A health status that prohibits physical exercise, such as unstable heart disease, severe systematic illness or severe orthopedic conditions; (2) if prolonging the preoperative period by 1–2 weeks was consistent with a medical risk; and (3) if the participant lived outside the catchment area of the included primary care units. The ethical application was approved by the Regional Ethical Board in Stockholm in September 2015 (Dnr: 2015/1179-31). An additional application including a higher intensity of the overall training intervention was approved in August 2016 (Dnr: 2016/1587-32). The trial was registered at ClinicalTrails.gov 08/09/2016, registration no. NCT02895464.

### Procedure

At the first visit in the surgical office, eligible participants received oral and written study information from a surgeon and nurse. The following day, a physiotherapist contacted the eligible participant by telephone for additional information and to ask for participation. Written informed consent was collected before the baseline assessments commenced. Participants were randomized to an intervention group receiving home-based preoperative physical exercise, or a standard care group. The sequence of allocation was generated through a web-based spreadsheet and conducted in blocks of five with six participants each. As a sample size calculation for a definitive trial was initially intended, the desired sample size was therefore 30 individuals [27]. However, due to a slow recruitment rate, coupled with the feasibility design and descriptive nature of primary outcomes, we ended the recruitment process earlier than intended [28]. The power calculation for the definitive trial will be based on a previous cohort study instead [29]. The study coordinator performed the randomization prior to the first assessment to enable surgical planning. Participants were unaware of their group allocation until after the first assessment, and assessors were blinded throughout three assessments of physical performance. It was, logically, not possible to blind study participants or physiotherapists conducting the exercise program in primary care due to the intervention type.

For both groups, two physiotherapists with expertise in the surgical field (with >20 years of clinical work experience each) conducted assessments of physical performance at the hospital at three time points: before the intervention, reassessments the day before surgery, and postoperative assessments on discharge from the hospital ward. Functional capacity was measured with the Six-Minute Walk Test according to guidelines from the American Thoracic Society statement [30], and walking distance in meters was recorded [31]. Habitual and maximal gait speed (m/s) were measured over 10 meters with a 2-meter acceleration and deceleration phase. Lower extremity strength was measured with the number of chair stands achieved during 30 seconds [32]. Inspiratory muscle strength was measured as maximal inspiratory pressure (MIP) with the respiratory pressure meter Micro RPM (Care Fusion, San Diego, California, USA) and registered as cmH_2_O. We developed an instruction manual and the physiotherapists were collectively trained before the study to assure consistent and standardized assessments. In addition, the physiotherapists were familiar with the assessment battery from a previous study [29]. The assessors and the physiotherapists conducting the exercise worked at separate locations and had no mutual contact during the study. For the intervention group, physiotherapists with experience in home-based rehabilitation (with clinical experience ranging from one to >20 years, however not specifically in oncological patients) led the exercise sessions in the participants’ home. Two physiotherapists each from four primary care units were trained theoretically in formal workshop form about the intervention in two one-day sessions.

### Patient demographic and clinical characteristics

Data regarding age, sex, tumor stage, type of surgery, and laboratory markers (albumin and hemoglobin) were retrieved from the medical records and from patient interviews. Any chemo or radiotherapy prior to surgery was registered. Comorbidity was documented according to the Charlson Comorbidity Index, which consists of 19 weighted diseases with a score from 1–6 depending on their association with 1-year mortality [33]. Perception of health-related quality of life was assessed preoperatively with the cancer-specific core questionnaire EORTC QLQ-C30 and an additional questionnaire ELD14 for older people [34, 35]. Higher scores indicate less mobility, more worries, and greater burden of illness, but better perceived global health status, family support, and maintenance of purpose [34, 35]. The Physical Activity Scale for Elderly was used to report level of preoperative physical activity. The scale contains perceptions of occupational, domestic, and leisure activities performed during the past seven days, as reported by the patient [36].

### Intervention

The 1-hour supervised sessions in the participants’ homes included three blocks. The starting dose of exercise was based on the baseline assessments of physical performance as assessed in hospital. Block I consisted of inspiratory muscle training (IMT) conducted with the handheld electronic device Power Breathe K3 (POWERbreathe, International Ltd, UK). The resistance started at 50% of maximal capacity for 30 breaths twice a day [37]. Progression was encouraged and monitored with the built-in single breath test (S-index) at each supervised sessions by the physiotherapist. The resistance was gradually adjusted to achieve a perceived exertion of 5–7 on the Borg CR-10 scale, where if the participant’s estimation was lower, the resistance was then increased by 5%. Block II consisted of high-intensity (perceived exertion of 7–8 on Borg CR-10) functional strength exercises such as chair stands and step-up with weight belts starting with 3x10 repetitions. The physiotherapist monitored progression with a chair-stand test at each session to establish the weight load and number of repetitions, which were increased if the estimation of exertion was lower than 7 on Borg CR-10 [38]. Block III consisted of endurance training such as bouts of stair climbing, Nordic walking outdoors, and interval walking indoors and/or outdoors. Intervals (perceived exertion of 7–8 on Borg CR-10) with, for example, brisk walking on a plain surface or with inclines in a small hill were conducted to increase intensity [17]. The number of steps was registered with a pedometer and interval duration was noted. The duration of the endurance session, as well as the number and length of the intervals were increased for progression. Blocks II and III were combined with functional task exercises according to each individual participant’s needs based on the Patient Specific Functional Scale (PSFS) [39, 40]. PSFS was used as an instrument to identify and facilitate functional activities for inclusion in the exercise intervention. The patients were required to define one to three activities of importance to them but difficult to perform. The activities were graded on a scale from 0 (“unable to perform activity”) to 10 (“able to perform activity without any restrictions”).

The training was conducted 2–3 times/week, for at least two weeks, with a minimum number of six supervised sessions or until surgery was due. On unsupervised days, participants were instructed to follow the recommendation of 150 minutes/week of moderate physical activity, do a core set of functional strength exercises (chair stands and step-up, but without weight belts) 2–3 times/week, and perform IMT for 30 breaths twice a day. All IMT sessions could be monitored in the Power Breath K3. Participants registered the occasions and dosage of self-administered training in an exercise diary.

The exercise protocol was used as a guideline; individual adjustments were made within the blocks if needed due to comorbidities such as rheumatoid arthritis or chronic obstructive pulmonary disease. Intensity was assessed and described by perceived exertion with the Borg CR-10 scale, which is a 10-point scale ranging from “nothing at all” to “maximal”, where 5 corresponds to the verbal anchor “strong” [31]. The Borg CR-10 scale was chosen due to the age-related decline in maximal heart rate, influence of medications (such as beta-blockers) or comorbidities limiting the ability to compare an objective measurement such as heart rate with subjective perceptive exertion [17, 41].

The standard care group followed the standard procedure of a two-week waiting period with ordinary preoperative information, and the advice to follow the recommendation of 150 minutes/week of moderate physical activity [42].

### Primary outcome measures (process feasibility)

The primary outcome was process feasibility (as described by Thabane et al. 2010) conducted with the variables recruitment rate, exercise compliance, and acceptability [25]. We defined recruitment rate as the number of participants included from the eligible patients, which was recorded on screening lists by the nurses at weekly CRC conferences. Compliance with the exercise intervention was defined as the number of sessions attended out of planned sessions and was registered in the exercise logs by the physiotherapists in primary care. Adherence rates (attended out of planned sessions) to exercise programs for older people were previously reported to range from 58–77%, whereas an adherence rate of >80% of supervised sessions was considered feasible in this study [43, 44]. Acceptability was determined using two surveys evaluating the participants’ and instructors’ satisfaction with the exercise intervention. The participant survey contained 19 questions while the instructor survey had 16 questions, with 1–4 or 1–3 response options arranged in an ordinal manner. Both questionnaires further allowed free-text answers.

### Secondary outcome measures (scientific feasibility)

The secondary outcomes were scientific feasibility, as described by Thabane et al. 2010, involving “the assessment of treatment safety, determination of dose levels and response, and estimation of treatment effect and its variance” [25]. The presence of adverse events during supervised sessions was registered in the exercise logs by the physiotherapist and in exercise diaries by study participants for the self-training. The events were divided into musculoskeletal-related, cardiovascular episodes, falls, and healthcare use [45]. Estimated treatment results were reported as median change from baseline to pre-surgical physical assessments within each group, and as effect size of the difference between groups.

On discharge, patient-reported recovery was collected with the 17-item Postoperative Recovery Profile (PRP) representing five subdomains (physical symptoms, physical function, psychological and social impact, and activity) [46]. A higher score indicates better recovery. Data on postoperative complications within the first 30 days after surgery were retrieved from the medical records according to the Clavien-Dindo grading system. The complications are categorized as infectious, neurological, cardiovascular, surgical, and other complications, on a scale from I–V [47]. Length of stay was defined as time spent in hospital from the day of surgery until participants were ready for discharge to their homes, rehabilitation clinic, or institutional care setting.

### Statistical analysis

Statistical analysis was performed using STATA SE (version 14.2; College Station, TX) and SPSS statistics (version 24.0; IBM Corp. Armonk, NY). Due to the small sample size, non-parametric tests were chosen when describing and analyzing data. Demographic and clinical data were reported as proportions, median with interquartile range, or 95% confidence intervals. Between-group analyses were performed with the Mann Whitney U-test for ordinal data and continuous data, and Fisher’s exact test for nominal data. The significance level was set at a p-value of ≤0.05. Primary outcomes were described in counts and frequencies. Within-group analyses regarding physical performance at baseline and repeated measures were performed with Friedmans ANOVA and a post-hoc test for pairwise comparison (reported with a Bonferroni-corrected alpha level 0.05/3 = 0.0167). To estimate the preliminary treatment effect and its variance in the difference between pre- and post-intervention measures between groups, effect size was calculated and reported as the Probabilistic Index (based on the Mann-Whitney statistics). It reports the probability that a given value in the intervention group is higher than a value in the standard care group [48].

## Results

**Fig 1:**
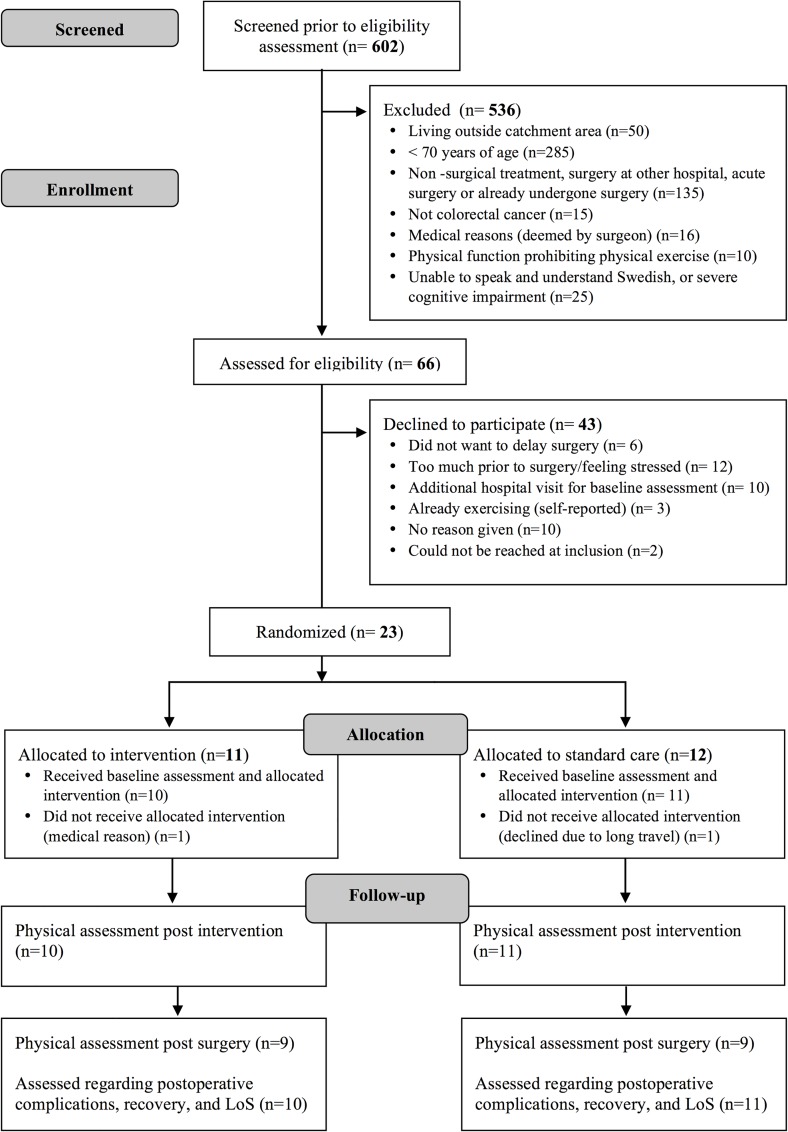
CONSORT flow diagram.

A total of 602 patients were screened for inclusion at the CRC conferences. Of these, only 66 met the inclusion criteria (median age 77 years (IQR 73–84); 47% males), see flow diagram (Fig 1). Of the 43 individuals declining participation, the median age was 77 years (IQR 74–83) and 49% were men, compared to a median age of 76 years (IQR 73–84) and 38% men in the study population (p = 0.67 for age, and p = 0.68 for sex). Of the 23 participants included, 11 were randomized to the intervention group and 12 to the standard care group. One participant per group was excluded after allocation due to medical reasons and transportation issues. Demographic and clinical characteristics of each group are reported in Table 1. The baseline characteristics of the intervention and standard care groups were comparable, except for an older age and more reported worries about the future in the intervention group (see Table 1).

**Table 1:** Demographic and clinical characteristics per allocation group.

Variable	Value			
	Total n = 21	Intervention n = 10	Standard care n = 11	*p-value*
Age, median (IQR)	76.0 (73–84)	83.5 (76–85)	74.0 (73–76)	**0.026**^a^
Sex, n (%)				
*Male*	8 (38)	4 (40)	4 (36)	0.608^b^
Living with partner, n (%)	10 (48)	5 (50)	5 (46)	0.590^b^
Cancer type, n (%)				0.538 ^b^
*Colon*	18 (86)	9 (90)	9 (82)	
*Rectal*	3 (14)	1 (10)	2 (18)	
Charlson Comorbidity Index, median (IQR)	1 (0–3)	2 (1–3)	1 (0–3)	0.425^a^
Smoking, n (%)				0.520^b^
*No*	10 (48)	4 (40)	6 (55)	
*Yes*	1 (4)		1 (9)	
*Previous smoker*	10 (48)	6 (60)	4 (36)	
Surgical approach, n (%)				0.633^b^
*Open*	6 (29)	3 (30)	3 (27)	
*Laparoscopic*	15 (71)	7 (70)	8 (73)	
Minutes of surgery, median (IQR)				
*Colon*	156 (140–176)	150 (140–164)	159 (145–176)	0.627^a^
*Rectal*	441 (401–616)	401	529 (441–616)	0.221^a^
Tumor stage, n (%)				0.052^b^
*0* ^*§*^	2 (9.5)		2 (18.2)	
*I*	3 (14.3)		3 (27.3)	
*II*	6 (28.6)	5 (50.0)	1 (9.1)	
*III*	9 (42.8)	4 (40.0)	5 (45.4)	
*IV*	1 (4.8)	1 (10.0)		
Neoadjuvant radiation, n (%)	3 (14)	1	2	0.538^b^
Laboratory markers, median (IQR)				
*Hemoglobin*, *g/L*	121 (111–130)	116 (108–126)	125 (113–133)	0.418^a^
*Albumin*, *g/L*	36 (33–38)†	36 (34–38)	35 (32–36)	0.277^a^
HRQoL domains, median (IQR)				
*Global health status (QLQ-30)*	66.7 (41.7–75)	45.84 (33.3–75)	66.7 (58.3–75)	0.453^a^
*Mobility (ELD14)*	22.3 (11–33.3)	27.8 (22.3–55.7)	22.3 (0–22.3)	0.138^a^
*Worries about others (ELD14)*	33.3 (16.7–50)	33.3 (16.7–50)	16.7 (0–33.3)	0.313^a^
*Future worries (ELD14)*	33.3 (22.3–55.7)	55.7 (33.3–66.7)	22.3 (22.3–33.3)	**0.034**^a^
*Maintaining purpose (ELD14)*	66.7 (33.3–83.3)	58.3 (33.3–83.3)	66.7 (33.3–83.3)	0.748^a^
*Burden of illness (ELD14)*	50 (33.3–66.7)	50 (33.3–66.7)	33.3 (16.7–50)	0.193^a^
*Family support (ELD14)*	66.7 (66.7–100)	83.3 (66.7–100)	66.7 (0–100)	0.495^a^
Self-reported physical activity, median (IQR)	73 (52–107)	72.5 (52–93)	76 (52–121)	0.573^a^

**Abbreviations:** IQR = interquartile range, HRQoL = Health-Related Quality of Life

^*§*^ = including colon (n = 2)

† = 19 due to missing data in medical records for two participants (both in the intervention group)

^a^ Mann-Whitney U test

^b^ Fisher exact test

### Process feasibility

#### Recruitment rate

Of 66 eligible patients, 23 were included, corresponding to a 35% recruitment rate. The most common reasons for declining participation were other time-consuming preoperative examinations; no time for the intervention; or feeling stressed prior to surgery (n = 12, 28%) (Fig 1). Ten (23%) individuals declined due to an extra hospital visit for baseline assessments, and six (14%) did not want to delay time until surgery.

#### Compliance with the exercise intervention

The median number of supervised sessions attended was 6 (range 4–8) out of a median of 6 planned sessions (range 5–8). Two participants missed one session each (one due to the physiotherapist being unable to conduct the last session, and one due to medical reasons resulting in rescheduling surgery). Hence, 58 sessions were performed out of 60 planned, corresponding to 97% compliance. Concerning the self-administered, unsupervised, training sessions, participants reported a median of 19 (IQR 18–22) IMT, 6 (IQR 2–9) functional strength and 8.5 (IQR 6–10) aerobic sessions.

#### Intervention acceptability

Participants in the intervention group overall responded with a high level of acceptability (using the agree and strongly agree response options) regarding factors such as information given and the content and meaningfulness of the intervention. Seven participants would have preferred a longer intervention period. The median number of days from inclusion in the study until surgery was 17 (range 14–24), and the preoperative period was prolonged with a median of 7 days (range 0–13) compared to the standardized care process of 14 days. All participants perceived the intensity level of the supervised exercise blocks to be satisfactory, and one participant perceived the intensity of the self-administered exercise as too high (Table 2). Overall, the instructors also responded with agree/strongly agree, except for one instructor perceiving difficulties in achieving high intensity and challenges with planning exercises. Three instructors found barriers with directly applying the intervention in their current clinical context, mainly due to time limitations as seen in the free text answers in Table 3.

**Table 2:** Participant satisfaction with the exercise intervention (n = 9, one participant did not respond).

		Participant response, n = 9				
Statement/Question						
Did you get enough information on the study?		Yes: 9			No: 0	
		Strongly disagree	Disagree	Agree	Strongly agree	Do not know
Did you get enough information from the instructor?					9	
Was the content of the supervised exercise relevant?				1	8	
Was the content of self-administered exercise relevant?				2	7	
I would have preferred to have a longer preoperative intervention period than currently possible:		1		3	4	1
I was able to push myself during the exercise:				6	3	
The intervention felt safe:				2	7	
It was motivating to exercise at/nearby my home:				1	8	
It was comfortable to exercise at/nearby my home:				2	7	
I would rather exercise at an out-patient clinic:		7	1			1
I found it meaningful to exercise before my surgery:				4	5	
I felt better prepared physically for my surgical treatment after the intervention:				5	3	1
		**Too low**	**Satisfactory**		**Too high**	
The level of inspiratory muscle training was:			9			
The level of aerobic endurance training was:			9			
The level of functional strength training was:			9			
The level of the self-administered training was:			8		1	
Support from next of kin		**Yes:** 7	**No:** 1		**N/A:** 1	
Overall satisfaction with participating in the study:	*“It was interesting to get exercise help from a specialist*. *It made me feel as I was being taken care of*, *and thus I have exercised more to show that I can*.*”* *“I live alone and have no relatives*, *so the social contact with the physiotherapist was invaluable in addition to the meaningful exercise*.*”* *“I’m feeling much stronger*, *like I gained muscles*, *and I got to work with my breathing at the highest level*. *Also*, *I can walk down stairs*, *which I was bad at before*.*”* *“It was positive to challenge myself and get a little breathless*. *It was interesting to be able to participate*, *and that I got help to improve my fitness*. *A very good coach/instructor*.*”* *“It was good/very good*.*”* ^**a**^ *“Meaningful*. *Unfortunately I was not able to complete the full program; however*, *I am happy that the surgery happened so soon*.*”* ^**b**^					
Suggestions for change?	*“If ethically allowed*, *I would like a longer exercise period i*.*e*. *further postponement of surgery*.*”* *“A longer exercise period would hopefully give even better results*.*”* *“Higher sound volume on the inspiratory muscle trainer*.*”*					

**Abbreviations:** N/A = Not applicable

^**a**^ Comment from three participants

^**b**^ Earlier surgical date due to anemia.

**Table 3:** Instructor satisfaction with the exercise intervention.

		Instructor response, n = 6				
Statement/Question						
Did you feel prepared before study start?		Yes: 6			No: 0	
		Strongly disagree	Disagree	Agree	Strongly agree	Do not know
The information from the hospital was sufficient to plan the exercise:				3	3	
The content of the intervention was relevant:				2	4	
The physical exercise was high intensive:			1	2	3	
I was able to challenge the participants:				4	2	
The participants were involved in exercise planning:			1	2	3	
The physical exercise was performed as planned:			1	4	1	
I had enough time to plan, conduct, and administer the exercise intervention:			1	4	1	
The study documents were easy to use and feasible:				3	3	
My competence was enough to conduct the exercise:				1	5	
The home-based approach was feasible:				1	5	
The intervention felt safe:					6	
Barriers:	*“The overall time aspect (two physiotherapists at each unit are needed)*. *The participants were sometimes too worried to focus fully on the exercise*.*”* *“Being able to conduct the planned number of exercise sessions is a challenge*, *both based on the resources of the primary care unit and the participants’ other commitments*.*”* *“The inspiratory muscle trainer can be difficult for some patients to use on their own*.*”* *“Time-consuming and at short notice*.”					
Possibilities:	*“Home-based exercise means good accessibility for the patients*. *Good opportunities to increase intensity and see improvement*.*”* *“Well-informed patient prior to surgery*, *less anxious and they may be able to initiate physical exercise faster postoperatively*. *Also*, *contact has already been initiated with the primary care unit*, *improving the postoperative care continuum*.*”* *“Increased amount of physical activity in the patients’ everyday life*.*”* *“Works well with patients who can go outside*, *but can also work okay for those who cannot go outside*. *Easy to adjust the intensity of the exercise*.*”* *“That the patient may continue to exercise after the surgery*.*”*					
Suggestions for change?	*“To shorten the visits*: *Conduct the IMT and functional strength exercise during the supervised visits and do the aerobic exercise within the self-administered exercise*. *Remove the PSFS*.*”* ^**a**^ *“Look through the Borg scale more in depth with the participant prior to the intervention*.*”* *“Even clearer instructions regarding the inspiratory muscle trainers*, *also look over the inspiratory muscle trainers one additional time before training to avoid technical issues*.*”* ^**a**^					

^**a**^ Comment from two instructors

### Secondary outcome—Scientific feasibility

#### Presence of adverse events

One participant reported joint pain due to existing knee arthritis, and another reported back and leg pain due to rheumatoid arthritis (and no cortisone use prior to surgery). One participant had episodes of dizziness during the aerobic exercise at three supervised sessions. No adverse events occurred during the self-administered training according to self-reported data from participants’ exercise diaries. No falls or additional healthcare use was reported during the intervention period, and none of the adverse events led to a manifest injury.

#### Dose and intensity of exercise intervention

The median intensity during the supervised sessions was achieved for IMT (at least 5 on the Borg CR-10), but was on average lower than the planned intensity for functional strength and aerobic endurance (7–8 on the Borg CR-10) (Table 4). The median IMT load achieved during the supervised sessions was 54% (range 47–80%) of maximal capacity. Nine out of ten participants used weight belts during the supervised functional strength exercises. Median weight loads and progression are reported in Table 4. Types of activities chosen for the patient-specific integration were jogging, making the bed, stair climbing, carrying groceries up the stairs, vacuum-cleaning, up-hill walking, swimming, walk a longer distance, and carrying wood from the garage (one participant did not choose a specific activity).

**Table 4:** Median intensity achieved during supervised and self-administered sessions, intervention group n = 10.

	Total	Session 1	Session 2	Session 3	Session 4	Session 5	Session 6
**Supervised exercise**							
	Intensity Borg CR-10, median (IQR)						
Exercise type							
*IMT*	5 (3–6)	3.5 (3–5)	4 (4–5)	4.5 (3.5–6)	5 (4–6)	5 (3–5)	6 (5–6)
*Chair rise*	6 (5–7)	5 (3–5)	6 (5–6)	6.5 (5–7)	6 (5–7)	6 (5–7)	6 (4–7)
*Step-up*	5 (4–6)	3.5 (2–4)	5 (5–6)	6 (5–6)	5 (5–6)	5 (5–7)	5 (4–7)
*Aerobic*	5 (5–7)	5 (4–5)	6.5 (5–7)	6 (5–7)	5 (5–7)	6 (5–7)	5 (5–7)
	Load weight belts (% of body weight), median (IQR)						
*Chair rise*	5 (4–6)	3.8 (3–5)	5.2 (4–5)	5.2 (4–6)	5.4 (5–7)	5.1 (5–7)	5.8 (4–9)
*Step-up*	5 (4–7)	3.8 (3–5)	5.1 (4–5)	5.2 (4–6)	5.4 (5–7)	6 (5–7.1)	7.1 (5–8)
	% of Maximal Inspiratory Pressure, median (IQR)						
*IMT*	54 (50–57)	50 (49–51)	51 (50–55)	53 (51–57)	55 (52–59)	55 (54–60)	56 (55–65)
**Self-administered exercise**							
	Intensity Borg CR-10, median (IQR)						
*IMT*	5 (4–5.5)						
*Functional strength*	5.5 (5–6)						
*Aerobic*	5 (5–6)						

**Abbreviations:** IQR = Interquartile range, IMT = Inspiratory Muscle Training

#### Estimation of treatment effect and its variance

At baseline, the intervention group had somewhat lower values on the physical performance tests (except for MIP) compared to the standard care group, as seen in Table 5. Between-group analyses revealed that the intervention group had a statistically significant increase from baseline to post-intervention assessment in MIP compared to the standard care group (p<0.01). The effect size analyses, regarding MIP, performed with the Probabilistic Index showed that the probability of an observation in the intervention group having a higher value than an observation in the standard care group was 90% (Table 5).

**Table 5:** Physical performance during the perioperative period within and between the intervention (n = 10) and standard care (n = 11) group.

	Intervention group						Standard care group						Between groups
	Baseline	Pre-surgery	Change		Post-surgery		Baseline	Pre-surgery	Change		Post-surgery		Change from baseline to pre- surgery
Variables	*Median* (95% CI)			*p*^§^^,^^a^		*p*^§^^,^^b^	*Median* (95% CI)			*p*^§^^,^^a^		*p*^§^^,^^b^	*ES (95% CI)*
Walking distance, meters	418.5	398	15	NS	330	**0.03**	432	426	-4	NS	278.5	**0.003**	0.64
	(300; 531)	(279; 553)	(-29; 46)		(241; 448)^†^		(357; 476)	(380; 482)	(-16; 20)		(115; 402)^††^		(0.35; 0.93)
Functional leg strength, n	11	13.5	+3.5	NS	11	NS	11	12	+1	NS	11.5	**0.02**	0.68
	(6; 14)	(10; 16)	(0; 4)		(1; 13)^†^		(8; 15)	(9; 16)	(-0.3; 3.3)		(4; 14)^††^		(0.41; 0.94)
Gait speed, m/s													
*Habitual*	1.04	1.15	+0.09	NS	0.89	**0.01**	1.06	1.12	+0.09	NS	0.80	NS	0.56
	(0.86; 1.25)	(0.91; 1.53)	(0; 0.3)		(0.71; 1.24)^†^		(0.86; 1.22)	(0.94; 1.26)	(-0.002; 0.13)		(0.60; 1.08)^††^		(0.25; 0.86)
*Maximal*	1.57	1.57	-0.02	NS	1.15	NS	1.67	1.56	0	NS	1.28	**0.04**	0.32
	(1.11; 1.91)	(1.01; 1.91)	(-0.1; 0.03)		(0.94; 1.50)^†^		(1.20; 1.89)	(1.29; 2.05)	(-0.1; 0.3)		(0.73; 1.44)^††^		(0.06; 0.59)
MIP, cmH_2_O	63.5	78	+17	**0.01**	79	NS	61	62	-2	NS	44	NS	0.90
	(37; 90)	(60; 111)	(7; 25)		(56; 89)^†^		(44; 89)	(44; 97)	(-6; 4)		(23; 79)^†^		(0.71; 1.08)*

**Abbreviations:** CI = Confidence Interval, MIP = Maximal Inspiratory Pressure, ES = Effect size (Probabilistic Index based on Mann Whitney U test [48]).

^†^ n = 9

^††^ n = 8

^§^ = Bonferroni-corrected p-value from pairwise comparison if the Friedmans ANOVA was statistically significant

^a^ = Between baseline and pre-surgery

^b^ = Between pre-surgery and post-surgery

NS = Not statistically significant

* p<0.01 (Mann Whitney U test)

**Fig 2:**
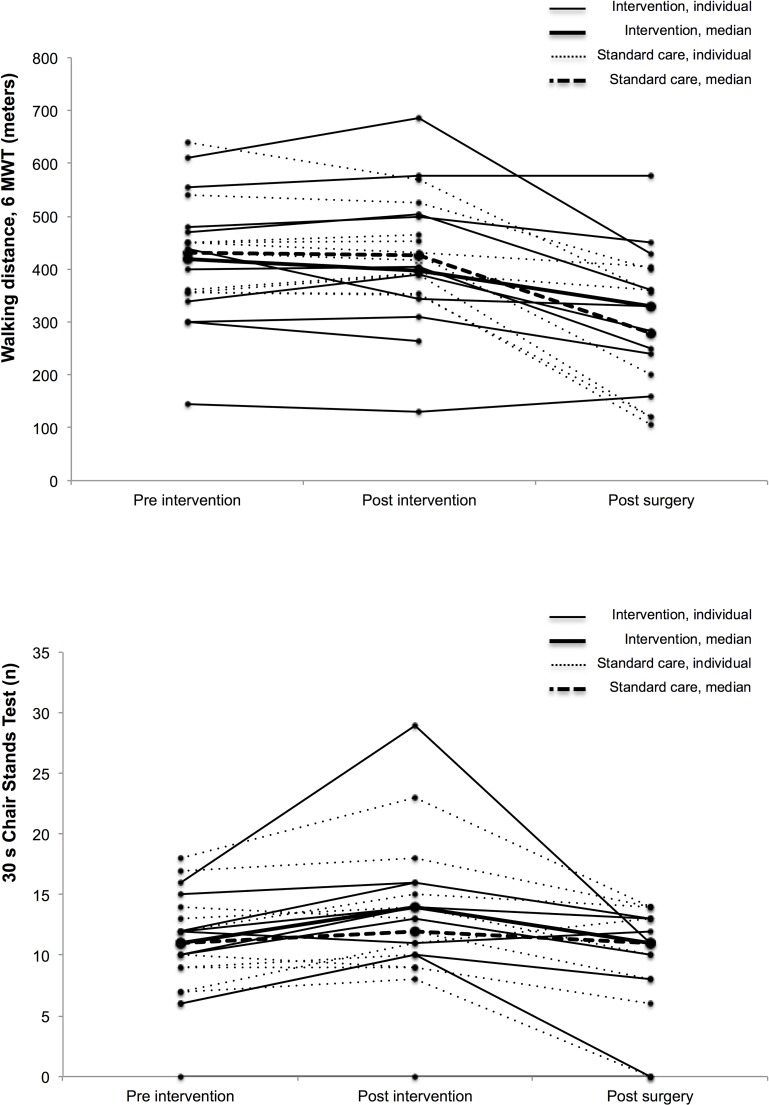
(a) Individual and median walking distance pre-, post-intervention and post surgery per group (b) Individual and mean number of chair stand during 30 sec pre-, post-intervention and post surgery per group.

In within-group analyses, the intervention group had a statistically significant preoperative improvement in MIP after the intervention, and declined significantly in walking distance and habitual gait speed post surgery. The standard care group did not improve significantly in any of the physical performance tests prior to surgery, and declined significantly in walking distance, leg strength, and maximal gait speed post surgery (Table 5). A large variability across individuals was seen in both groups (Fig 2).

Postoperative complications were observed in eight participants (38%); however, none of these were higher than a Clavien-Dindo grade II (“requiring pharmacological treatment with drugs” [47]). A higher number of complications, shorter median LoS, and better patient-reported recovery were observed in the intervention compared to the standard care group. However, no differences reached statistical significance (Table 6).

**Table 6:** Postoperative outcomes of intervention group (n = 10) versus standard care group (n = 11).

	Intervention group	Standard care group		
			*p*	*ES (95% CI)*
Outcome				
Any postoperative complications, n (%)	6 ^a^	2 ^b^	0.06^§^	-
Length of stay, median days (IQR)	5 (4–6)	6 (4–7)	0.57^§§^	0.43 (0.15; 0.71)
Patient-reported recovery, median (IQR)	5.5 (2–10)	2 (0–5)	0.22^§§^	0.67 (0.36; 0.97)

Type of complications

^a^ Two wound infections, one pneumonia, two urinary tract infections, and one pulmonary embolism

^b^ One disturbance of fluid balance, and one wound infection.

^§^ Fisher exact test

^§§^ Mann-Whitney U test

## Discussion

We undertook this feasibility study to evaluate the process for a future larger scale trial with the main questions described by Thabane et al. 2010: Can it be done? / Should it be done? / And if so, how? We found that supervised home-based physical exercise in one-third of the total fraction of eligible persons, prior to colorectal cancer surgery, seems feasible with respect to compliance and acceptability of the intervention. However, low recruitment is a challenge, which might result in selection bias, as the feasibility of the exercise training is unknown in the non-recruited people. Consequently, modifications are suggested for a larger scale trial to improve, for example, recruitment rates and methods to increase intensity.

Like previous studies, the primary issue of feasibility was the low recruitment rate. The low recruitment can in turn lead to selection bias, where consequences of selection-bias are threats to external validity and generalizability of study findings [49]. This should be taken into consideration when interpreting the results. The target population of the study is considered a vulnerable group based on comorbidities and malignancy. It has previously been noted that the number needed to screen for inclusion of an older participant is 1:3 [18]. In a study that interviewed older people who declined participation in an exercise trial, barriers mentioned were related to being active already, poor health, psychosocial factors, and lack of time. Facilitators to increase recruitment included tailored trial information and evidence that physical exercise is beneficial [50]. Of the eligible participants in this study, the median age and sex distribution were similar when comparing those who declined with included individuals. It would have been valuable to include physical fitness in the comparison, as patients with poor mobility decline to a larger extent, which could result in bias arising from self-selection [51]. Nevertheless, data was not available for patients who declined to participate in our study.

In relation to the above stated, we need to discuss how to improve recruitment for the definitive trial. Almost half of the screened patients were not eligible for this study due to an age ≤70 years. A revision of eligibility criteria such as basing the preoperative screening on physical performance rather than chronological age to target high-risk patients was previously recommended and may include a larger age span [29]. Two out of three eligible patients declined participation. Reasons to decline included risk of delaying surgery and feelings of stress due to other time-consuming preoperative examinations, for example. This may partly be connected to preoperative time constraints in colorectal cancer care, as well as increased anxiety due to the cancer diagnosis and the upcoming surgery. Nevertheless, the preoperative psychological state is highly variable between individuals and was not investigated in depth in this study. The aim of the new standardized care processes in Sweden, which are national quality guidelines, is to diminish national differences in waiting time and decrease patient distress. However, it is also stated that there is no evidence that a 1–2 week difference in waiting time affects survival [2, 52, 53]. It is vital to communicate this information to eligible patients, as not wanting to delay surgery was an important reason for declining. Nevertheless, the evidence regarding the effects of preoperative physical exercise is not yet robust enough to motivate prolonging the preoperative period from a risk-benefit point of view. This must be further discussed in a multidisciplinary manner. Feeling stressed prior to surgery can also be related to fatigue and/or reduced physical capacity, meaning that we also need to consider how to approach the more frail patients preoperatively with clear and tailored information. For example, avoid phrases such as “delaying surgery” and focus on the preoperative “preparation” period instead. Ten patients declined due to an extra hospital visit for baseline assessments; an alternative option is to conduct base assessments in participants’ homes or organize transport to facilitate participation. In combination with suggested improvements for a definitive trial, a multicenter approach should be considered to expand the catchment area and the number of eligible patients, as well as other options for alternative designs as was recommended in a recent review [54].

Compliance in the supervised sessions was high in the participating fraction of patients and it exceeded the feasibility goal of >80%. It should be kept in mind that this could also be related to the highly possible selection bias in this study, as program participants might have been more interested in physical activity than non-participants, a phenomenon seen more often and necessary to be dealt with in the trial we are planning [51]. Previous literature reports a large variability (16 to 96%) in compliance rates to exercise programs prior to colorectal cancer surgery [6, 7]. Issues with low exercise compliance are likely to inhibit the effects on outcomes. One review investigating predictors of exercise adherence during/after cancer treatment (not pre-surgical only) stated that one of the most important factors was related to where the intervention was located [20]. Hence, one strength of this study is the supervised home-based approach (in combination with self-administered exercise), which increased compliance and added the ability to conduct the exercise in the older person’s daily environment. Home-based preoperative exercise has been shown to be feasible prior to orthopedic surgery [21], cardiac surgery [55], lung cancer surgery [56], and pancreatic surgery [57]. However, previous studies of home-based exercise are mainly self-administered as opposed to combining it with supervised home-based exercise. Compliance appears to be higher in supervised exercise interventions [43]. Compliance with the supervised sessions is easy to register, but compliance with self-administered exercises, as an adjunct to supervised sessions, should be interpreted with caution due to self-reporting, which may generate a bias by either over- or underestimating exercise dose. In this study, for titration, we monitored the progress of the fitness parameters as described in the intervention paragraph. Another possibility is to use technologies such as accelerometers to reduce reporting bias in self-administered exercise.

The exercise program fulfills the items of the CONTENT scale concerning rationale, content and adherence [16]. However, two items of the scale should be further discussed in relation to this study. First, as previously mentioned, there is a risk of selection bias. This must be considered when interpreting the applicability of the feasibility outcomes. Second, the physiotherapist in primary care had no specific experience with patients with colorectal cancer. In communication with instructors who conducted the exercise, multiple participants mentioned that the intervention was easier to conduct and less time-consuming as the exercise protocol became more familiar. Preoperative exercise in patients with colorectal cancer has not yet been implemented in Swedish primary care. Hence, it is important for physiotherapists conducting the preoperative exercise to expand their experience with these types of patients and training. It is also important to consider that some components of the supervised sessions, such as monitoring of heart rate and saturation, are related to research context and might be reduced if implemented in the clinic.

Overall, participants found the intervention acceptable regarding type, dose, and intensity. They addressed the valuable aspects of receiving guidance from, and having social interaction with, a physiotherapist. The instructors recognized the potential benefits of increased physical activity levels overall for participants of the program during the perioperative period. One challenge pointed out was the preoperative time constraint previously discussed in this article. Another challenge was difficulties in achieving higher exercise intensities. This was also seen in participants not achieving the planned intensity in perceived exertion during the blocks of functional strength and aerobic endurance. One instructor stated that it was sometimes hard for participants to make an adequate estimation on the Borg scale, with them tending to underestimate themselves [58]. Also, a progression was observed in both weight loads used during functional strength exercises and IMT (Table 4). Sufficient instructions and education of both participants and instructors are important prior to the intervention to enable participants to achieve higher intensities across different exercise modalities.

The type and dose of exercise in our study seemed to be safe, with a low rate of adverse events reported (most connected to already existing diseases, and none being severe). For high-risk patients, it might not be safe to maintain the same intensity during the self-administered sessions in comparison to the supervised session with the physiotherapist. One participant was excluded after randomization and one could not complete the last session as the planned surgery was performed earlier than scheduled due to anemia. Anemia, iron deficiency, and preoperative blood management are important aspects that need to be further discussed as they affect aspects such as oxygen transportation, functional status and postoperative outcomes [59]. For this feasibility study, we followed standard preoperative nutritional care (according to the ERAS guidelines for perioperative care in colorectal surgery [60] with a modified Subjective Global Assessment–SGA). A contact nurse screened the patient regarding e.g. BMI, changes in weight and appetite at the first visit to the surgical office. Contact with a dietician was initiated if a risk for malnutrition appeared. The dietician conducted a registration of dietary intake, gave individual diet advice, and prescribed supplements if needed.

We only aimed to report preliminary estimates and variability of the estimates in this study, as in pilot studies the aim is not to resolve ‘effectiveness’ but feasibility. Notably, we found that participants did respond to the intervention (but with a large individual variability). However, only inspiratory muscle strength improved significantly post intervention, and no significant loss was seen regarding inspiratory muscle strength on discharge in the intervention group. One possible reason may be that participants achieved sufficient intensity in the IMT block, but not for the other blocks. Incorporating short bouts of higher intensity intervals has previously been suggested as a way of increasing effectiveness in preoperative exercise interventions [17]. Another reason might be that the IMT is similar to the MIP used at the assessments.

In addition, the standard care group did not improve significantly in any of the physical performance tests prior to surgery, and declined significantly in walking distance, leg strength, and maximal gait speed post surgery. When interpreting the reported data, it should also be considered that the intervention group was significantly older, and had higher scores on the Charlson Comorbidity Index, which could be possible reasons for a somewhat lower baseline physical performance compared to the standard care group. In contrast, the intervention group reported more worries about the future at baseline than the standard care group and had a higher rate of postoperative complications, probably affected by an insufficient sample size, but also due to higher age and comorbidities. Nevertheless, they had a shorter median LoS and reported a better postoperative recovery compared to the standard care group. One possible reason, which needs further investigation, could be the social contact and support in guiding the exercise sessions provided by the physiotherapist during the supervised sessions, which may have strengthened participants’ self-efficacy. Moreover, with a larger sample size in a definitive trial, the randomization process should minimize differences in baseline data between the groups.

To conclude, suggested modifications for our future definitive trial are as follow:

- Use a multicenter approach to increase the number of eligible patients, and more extensive and tailored trial information at inclusion, for example changing the semantics such as “prolonging the waiting period”to focus on the preoperative “preparation”period instead in the patient information leaflets.
- Emphasize preoperative physical performance in preoperative screening, by adding tests of physical performance such as gait speed, functional leg strength, or functional physical capacity to conventional risk screening, rather than just chronological age to target high-risk patients.
- Conduct home-based baseline assessment or improve synchronization with already scheduled visits to the hospital to eliminate additional traveling for participants.
- Modify the exercise protocol to facilitate the daily work of the instructing physiotherapists in primary care, as well as consider portable tracking devices to register the self-administered exercise between supervised visits.
- Extend the education period for the instructing physiotherapists in primary care.
- To achieve treatment effects within the current time frame of only two to three weeks, exercise blocks should be of high intensity.

## Conclusions

We found that a short-term preoperative supervised home-based physical exercise intervention at a high level of estimated exertion can be conducted, with respect to compliance and acceptability, in older people with similar physical status as the study population, prior to colorectal cancer surgery. However, we suggest certain modifications for a definitive trial to improve recruitment rates and intensity. As surgery affects multiple organ systems, we need to involve multiple exercise domains in the interventions, such as IMT, strength training, and aerobic training. Furthermore, the heterogeneity in older adults’ health status is something we need to consider when designing an exercise intervention. A person-centered approach as well as the ability to modify exercise programs is needed due to different trajectories in aging. The possible effectiveness of physical exercise in connection with the current time restraints in preoperative cancer care needs to be further evaluated in larger trials.

## Supporting information

S1 Checklist CONSORT extension pilot and feasibility. (DOC)

S1 File English translation—Ethical protocol and approval. (PDF)

S2 File Swedish original—Ethical protocol and approval. (PDF)

## References

[1] Brenner H, Kloor M, Pox CP. Colorectal cancer. The Lancet. 2014;383(9927):1490–502. 10.1016/S0140-6736(13)61649-9 24225001

[2] The Swedish Regional Cancer Centres. Colon cancer: National quality report 2016 from the Swedish colorectal cancer registry [Internet]. Umeå: The Swedish Regional Cancer Centres; 2017 [cited 2018 3 Dec]. Available from: https://www.cancercentrum.se/globalassets/cancerdiagnoser/tjock—och-andtarm-anal/kvalitetsregister/rapporter-2017/kolon2016.pdf.

[3] Laghi F, Tobin MJ. Disorders of the respiratory muscles. Am J Respir Crit Care Med. 2003;168(1):10–48. 10.1164/rccm.2206020 12826594

[4] Griffiths R, Beech F, Brown A, Dhesi J, Foo I, Goodall J, et al Peri-operative care of the elderly 2014: Association of Anaesthetists of Great Britain and Ireland. Anaesthesia. 2014;69 Suppl 1:81–98. 24303864 10.1111/anae.12524

[5] Silver JK, Baima J. Cancer prehabilitation: an opportunity to decrease treatment-related morbidity, increase cancer treatment options, and improve physical and psychological health outcomes. Am J Phys Med Rehabil. 2013;92(8):715–27. 10.1097/PHM.0b013e31829b4afe 23756434

[6] Boereboom C, Doleman B, Lund JN, Williams JP. Systematic review of pre-operative exercise in colorectal cancer patients. Tech Coloproctol. 2016;20(2):81–9. 10.1007/s10151-015-1407-1 26614304

[7] Bruns ER, van den Heuvel B, Buskens CJ, van Duijvendijk P, Festen S, Wassenaar EB, et al The effects of physical prehabilitation in elderly patients undergoing colorectal surgery: a systematic review. Colorectal Dis. 2016;18(8):O267–77. 10.1111/codi.13429 27332897

[8] Barberan-Garcia A, Ubre M, Roca J, Lacy AM, Burgos F, Risco R, et al Personalised Prehabilitation in High-risk Patients Undergoing Elective Major Abdominal Surgery: A Randomized Blinded Controlled Trial. Ann Surg. 2018;267(1):50–6. 10.1097/SLA.0000000000002293 28489682

[9] Heldens AF, Bongers BC, de Vos-Geelen J, van Meeteren NL, Lenssen AF. Feasibility and preliminary effectiveness of a physical exercise training program during neoadjuvant chemoradiotherapy in individual patients with rectal cancer prior to major elective surgery. Eur J Surg Oncol. 2016;42(9):1322–30. 10.1016/j.ejso.2016.03.021 27156145

[10] Valkenet K, Trappenburg JC, Schippers CC, Wanders L, Lemmens L, Backx FJ, et al Feasibility of Exercise Training in Cancer Patients Scheduled for Elective Gastrointestinal Surgery. Dig Surg. 2016;33(5):439–47. 10.1159/000445958 27193943

[11] Dronkers JJ, Lamberts H, Reutelingsperger IMMD, Naber RH, Dronkers-Landman CM, Veldman A, et al Preoperative therapeutic programme for elderly patients scheduled for elective abdominal oncological surgery: a randomized controlled pilot study. Clin Rehabil. 2010;24:614–22. 10.1177/0269215509358941 20530651

[12] Minnella EM, Bousquet-Dion G, Awasthi R, Scheede-Bergdahl C, Carli F. Multimodal prehabilitation improves functional capacity before and after colorectal surgery for cancer: a five-year research experience. Acta Oncol. 2017;56(2):295–300. 10.1080/0284186X.2016.1268268 28079430

[13] Soares SM, Nucci LB, da Silva MM, Campacci TC. Pulmonary function and physical performance outcomes with preoperative physical therapy in upper abdominal surgery: a randomized controlled trial. Clin Rehabil. 2013;27(7):616–27. 10.1177/0269215512471063 23405020

[14] Pouwels S, Stokmans RA, Willigendael EM, Nienhuijs SW, Rosman C, van Ramshorst B, et al Preoperative exercise therapy for elective major abdominal surgery: a systematic review. Int J Surg. 2014;12(2):134–40. 10.1016/j.ijsu.2013.11.018 24325942

[15] Hijazi Y, Gondal U, Aziz O. A systematic review of prehabilitation programs in abdominal cancer surgery. Int J Surg. 2017;39:156–62. 10.1016/j.ijsu.2017.01.111 28161527

[16] Hoogeboom TJ, Oosting E, Vriezekolk JE, Veenhof C, Siemonsma PC, de Bie RA, et al Therapeutic validity and effectiveness of preoperative exercise on functional recovery after joint replacement: a systematic review and meta-analysis. PLoS One. 2012;7(5):e38031 10.1371/journal.pone.0038031 22675429 PMC3364996

[17] Weston M, Weston KL, Prentis JM, Snowden CP. High-intensity interval training (HIT) for effective and time-efficient pre-surgical exercise interventions. Perioper Med (Lond). 2016;5:2. 26770671 10.1186/s13741-015-0026-8 PMC4712564

[18] McMurdo ME, Roberts H, Parker S, Wyatt N, May H, Goodman C, et al Improving recruitment of older people to research through good practice. Age Ageing. 2011;40(6):659–65. 10.1093/ageing/afr115 21911335

[19] Ashworth NL, Chad KE, Harrison EL, Reeder BA, Marshall SC. Home versus center based physical activity programs in older adults. Cochrane Database Syst Rev. 2005(1):CD004017 10.1002/14651858.CD004017.pub2 15674925 PMC6464851

[20] Ormel HL, van der Schoot GGF, Sluiter WJ, Jalving M, Gietema JA, Walenkamp AME. Predictors of adherence to exercise interventions during and after cancer treatment: A systematic review. Psychooncology. 2018;27(3):713–24. 10.1002/pon.4612 29247584 PMC5887924

[21] Oosting E, Jans MP, Dronkers JJ, Naber RH, Dronkers-Landman CM, Appelman-de Vries SM, et al Preoperative home-based physical therapy versus usual care to improve functional health of frail older adults scheduled for elective total hip arthroplasty: a pilot randomized controlled trial. Arch Phys Med Rehabil. 2012;93(4):610–6. 10.1016/j.apmr.2011.11.006 22365481

[22] Ferreira V, Agnihotram RV, Bergdahl A, van Rooijen SJ, Awasthi R, Carli F, et al Maximizing patient adherence to prehabilitation: what do the patients say? Support Care Cancer. 2018;26(8):2717–23. 10.1007/s00520-018-4109-1 29478189

[23] de Vreede PL SM, van Meeteren NLU, Duursma SA, Verhaar HJJ. Functional-Task Exercise Versus Resistance Strength Exercise to Improve Daily Function in Older Women: A Randomized, Controlled Trial. JAGS. 2005;53(1):2–10. 10.1111/j.1532-5415.2005.53003.x 15667369

[24] Eldridge SM, Lancaster GA, Campbell MJ, Thabane L, Hopewell S, Coleman CL, et al Defining Feasibility and Pilot Studies in Preparation for Randomised Controlled Trials: Development of a Conceptual Framework. PLoS One. 2016;11:3. 10.1371/journal.pone.0150205 PMC4792418 26978655

[25] Thabane L, Ma J, Chu R, Cheng J, Ismaila A, Rios L, et al A tutorial on pilot studies: the what, why and how. BMC Medical Research Methodology. 2010;10:1 10.1186/1471-2288-10-1 20053272 PMC2824145

[26] Morgan B, Hejdenberg J, Hinrichs-Krapels S, Armstrong D. Do feasibility studies contribute to, or avoid, waste in research? PLoS One. 2018;13(4):e0195951 10.1371/journal.pone.0195951 29684043 PMC5912740

[27] Browne RH. On the use of a pilot sample for sample size determination. Statistics in medicine. 1995;14:1933–40. 8532986 10.1002/sim.4780141709

[28] Julious SA. Sample size of 12 per group rule of thumb for a pilot study. Pharmaceutical Statistics. 2005;4(4):287–91.

[29] Karlsson E, Egenvall M, Farahnak P, Bergenmar M, Nygren-Bonnier M, Franzen E, et al Better preoperative physical performance reduces the odds of complication severity and discharge to care facility after abdominal cancer resection in people over the age of 70—A prospective cohort study. Eur J Surg Oncol. 2018;44:1760–7. 10.1016/j.ejso.2018.08.011 30201418

[30] American Thoracic Society. ATS Statement: Guidelines for the Six-Minute Walk Test. Am J Respir Crit Care Med. 2002;166:111–7. 10.1164/ajrccm.166.1.at1102 12091180

[31] Borg GA. Psychophysical bases of perceived exertion. Med Sci Sports Exerc. 1982;14(5):377–81. 7154893

[32] Jones CJ, Rikli RE, Beam WC. A 30-s chair-stand test as a measure of lower body strength in community-residing older adults. Res Q Exerc Sport. 1999;70(2):113–9. 10.1080/02701367.1999.10608028 10380242

[33] Charlson ME, Pompei P, Ales KL, MacKenzie CR. A new method of classifying prognostic comorbidity in longitudinal studies: development and validation. J Chron Dis. 1987;40(5):373–83. 3558716 10.1016/0021-9681(87)90171-8

[34] Aaronson N, Ahmedzai S, Bergman B, Bullinger M, Cull A, Duez N, et al The European organization for research and treatment of cancer QLQ-C30: A quality-of-life instrument for use in international clinical trials in oncology. J Natl Cancer Inst. 1993;85(5):365–76. 10.1093/jnci/85.5.365 8433390

[35] Wheelwright S, Darlington AS, Fitzsimmons D, Fayers P, Arraras JI, Bonnetain F, et al International validation of the EORTC QLQ-ELD14 questionnaire for assessment of health-related quality of life elderly patients with cancer. Br J Cancer. 2013;109(4):852–8. 10.1038/bjc.2013.407 23868003 PMC3749575

[36] Washburn RA. Assessment of physical activity in older adults. Res Q Exerc Sport. 2000;71 Suppl 2:79–87. 25680017 10.1080/02701367.2000.11082790

[37] Bailey SJ, Romer LM, Kelly J, Wilkerson DP, DiMenna FJ, Jones AM. Inspiratory muscle training enhances pulmonary O2 uptake kinetics and high-intensity exercise tolerance in humans. J Appl Physiol. 2010;109(2):457–68. 10.1152/japplphysiol.00077.2010 20507969

[38] Glasziou P, Irwig L, Mant D. Monitoring in chronic disease: a rational approach. BMJ. 2005;330(7492):644–8. 10.1136/bmj.330.7492.644 15774996 PMC554914

[39] Horn KK, Jennings S, Richardson G, Vliet DV, Hefford C, Abbott JH. The patient-specific functional scale: psychometrics, clinimetrics, and application as a clinical outcome measure. J Orthop Sports Phys Ther. 2012;42(1):30–42. 10.2519/jospt.2012.3727 22031594

[40] Rosengren J, Brodin N. Validity and reliability of the Swedish version of the Patient Specific Functional Scale in patients treated surgically for carpometacarpal joint osteoarthritis. J Hand Ther. 2013;26(1):53–61. 10.1016/j.jht.2012.10.007 23195850

[41] Bray NW, Smart RR, Jakobi JM, Jones GR. Exercise prescription to reverse frailty. Applied Physiology, Nutrition, and Metabolism. 2016;41(10):1112–6. 10.1139/apnm-2016-0226 27649859

[42] Chodzko-Zajko WJ, Proctor DN, Fiatarone Singh MA, Minson CT, Nigg CR, Salem GJ, et al American College of Sports Medicine position stand. Exercise and physical activity for older adults. Med Sci Sports Exerc. 2009;41(7):1510–30. 10.1249/MSS.0b013e3181a0c95c 19516148

[43] Picorelli AM, Pereira LS, Pereira DS, Felicio D, Sherrington C. Adherence to exercise programs for older people is influenced by program characteristics and personal factors: a systematic review. J Physiother. 2014;60(3):151–6. 10.1016/j.jphys.2014.06.012 25092418

[44] Kampshoff CS, van Mechelen W, Schep G, Nijziel MR, Witlox L, Bosman L, et al Participation in and adherence to physical exercise after completion of primary cancer treatment. Int J Behav Nutr Phys Act. 2016;13(1):100 10.1186/s12966-016-0425-3 27612561 PMC5016937

[45] Liu CJ, Latham N. Adverse events reported in progressive resistance strength training trials in older adults: 2 sides of a coin. Arch Phys Med Rehabil. 2010;91(9):1471–3. 10.1016/j.apmr.2010.06.001 20801270

[46] Allvin R, Ehnfors M, Rawal N, Svensson E, Idvall E. Development of a questionnaire to measure patient-reported postoperative recovery: content validity and intra-patient reliability. J Eval Clin Pract. 2009;15(3):411–9. 10.1111/j.1365-2753.2008.01027.x 19366398

[47] Dindo D, Demartines N, Clavien P-A. Classification of Surgical Complications. Ann Surg. 2004;240(2):205–13. 10.1097/01.sla.0000133083.54934.ae 15273542 PMC1360123

[48] Conroy RM. What hypotheses do “nonparametric” two-group tests actually test? The Stata Journal. 2012;12(2):182–90.

[49] Slack MK, Draugalis JR. Establishing the internal and external validity of experimental studies. Am J Health-Syst Pharm. 2001;58:2173–84. 11760921

[50] Rogers A, Harris T, Victor C, Woodcock A, Limb E, Kerry S, et al Which older people decline participation in a primary care trial of physical activity and why: insights from a mixed methods approach. BMC Geriatrics. 2014;14:46 10.1186/1471-2318-14-46 24725730 PMC3991893

[51] Provencher V, Mortenson WB, Tanguay-Garneau L, Belanger K, Dagenais M. Challenges and strategies pertaining to recruitment and retention of frail elderly in research studies: a systematic review. Arch Gerontol Geriatr. 2014;59(1):18–24. 10.1016/j.archger.2014.03.006 24745811

[52] Curtis NJ, West MA, Salib E, Ockrim J, Allison AS, Dalton R, et al Time from colorectal cancer diagnosis to laparoscopic curative surgery-is there a safe window for prehabilitation? Int J Colorectal Dis. 2018;33(7):979–83. 10.1007/s00384-018-3016-8 29574506

[53] Hangaard Hansen C, Gogenur M, Tvilling Madsen M, Gogenur I. The effect of time from diagnosis to surgery on oncological outcomes in patients undergoing surgery for colon cancer: A systematic review. Eur J Surg Oncol. 2018;44(10):1479–85. 10.1016/j.ejso.2018.06.015 30251641

[54] Golfam M, Beall R, Brehaut J, Saeed S, Relton C, Ashbury FD, et al Comparing alternative design options for chronic disease prevention interventions. Eur J Clin Invest. 2015;45(1):87–99. 10.1111/eci.12371 25388015

[55] Waite I, Deshpande R, Baghai M, Massey T, Wendler O, Greenwood S. Home-based preoperative rehabilitation (prehab) to improve physical function and reduce hospital length of stay for frail patients undergoing coronary artery bypass graft and valve surgery. J Cardiothorac Surg. 2017;12(1):91 10.1186/s13019-017-0655-8 29073924 PMC5658994

[56] Coats V, Maltais F, Simard S, Fréchette É, Trembley L, Ribiero F, et al Feasibility and effectiveness of a home-based exercise training program before lung resection surgery. Can Respir J. 2013;20(2):e10–6. 10.1155/2013/291059 23616972 PMC3630051

[57] Ngo-Huang A, Parker NH, Wang X, Petzel MQB, Fogelman D, Schadler KL, et al Home-based exercise during preoperative therapy for pancreatic cancer. Langenbecks Arch Surg. 2017;402(8):1175–85. 10.1007/s00423-017-1599-0 28710540 PMC8372869

[58] Nelson ME, Rejeski WJ, Blair SN, Duncan PW, Judge JO, King AC, et al Physical activity and public health in older adults: recommendation from the American College of Sports Medicine and the American Heart Association. Med Sci Sports Exerc. 2007;39(8):1435–45. 10.1249/mss.0b013e3180616aa2 17762378

[59] Munoz M, Acheson AG, Auerbach M, Besser M, Habler O, Kehlet H, et al International consensus statement on the peri-operative management of anaemia and iron deficiency. Anaesthesia. 2017;72(2):233–47. 10.1111/anae.13773 27996086

[60] Gustafsson UO, Scott MJ, Hubner M, Nygren J, Demartines N, Francis N, et al Guidelines for Perioperative Care in Elective Colorectal Surgery: Enhanced Recovery After Surgery (ERAS) Society Recommendations: 2018. World J Surg. 2019;43(3):659–95. 10.1007/s00268-018-4844-y 30426190

