# Supplementary material for: Feasibility of preoperative supervised home-based exercise in older adults undergoing colorectal cancer surgery – A randomized controlled design
Source: PLoS One. 2019 Jul 2;14(7):e0219158. doi: 10.1371/journal.pone.0219158 (PMC6605852; doi:10.1371/journal.pone.0219158)
Supplement: S1 File — (PDF) [file pone.0219158.s002.pdf]

Note: This is a translation from the original language Swedish to English of the ethical application for the **full project,** in which this feasibility study is a sub-study. The text within boxes in the original Swedish document is general instructions for how to fill in the application and have therefore not been translated.

Filename: S2 Swedish original - Ethical protocol and approval.pdf

APPLICATION FOR ETHICAL REVIEW OF RESEARCH INCLUDING HUMANS

To the Regional Ethical Board in: Stockholm

Project title: Preoperative exercise to decrease the risk for complications and reduce length of stay in hospital in vulnerable older people undergoing abdominal- and thoracic surgery.

The application refers to: Research with one entity principally responsible for the research.

1. Information regarding entity responsible for the research etc

1:1 Entity principally responsible for the research
Name: Karolinska Institutet, Department of Neurobiology, Care Sciences and Society
Address: Alfred Nobels Allé 23, D3, 14183 Huddinge

1:2 Qualified representative for the entity responsible for the research
Name: Maria Eriksdotter
Position: Head of the Department of Neurobiology, Care Sciences and Society
Address: Karolinska Institutet, Department of Neurobiology, Care Sciences and Society, Alfred Nobels Allé 23, D3, 14183 Huddinge

1:3 Researcher responsible for conducting the project (contact person, Principal Investigator, PI)
Name: Elisabeth Rydwik
Position: Senior lecturer, Associate Professor, Registered Physiotherapist
Address: Karolinska Institutet, Department of Neurobiology, Care Sciences and Society, Alfred Nobels Allé 23, D3, 14183 Huddinge
E-mail: Elisabeth.rydwik@ki.se

1:4 Co-applicants
Erika Franzén, Associate Professor, Registered Physiotherapist. Karolinska University Hospital, Function Allied Health Professionals; Karolinska Institutet, Department of Neurobiology, Care Sciences and Society, Division of Physiotherapy.

Åsa Dedering, Associate Professor, Registered Physiotherapist. Head of Function Allied Health Professionals, Karolinska University Hospital.

Malin Nygren-Bonnier, PhD, Registered Physiotherapist. Karolinska University Hospital, Function Allied Health Professionals; Karolinska Institutet, Department of Neurobiology, Care Sciences and Society, Division of Physiotherapy.

Mia Bergenmar , Associate Professor, University nurse, Center for Digestive Diseases, Karolinska University Hospital, and Department of Oncology-Pathology, Karolinska Institutet

Bengt Isaksson, Associate Professor, MD, Center for Digestive Diseases, Karolinska University Hospital

Monika Egenvall, PhD, MD, Center for Digestive Diseases, Karolinska University Hospital

Parastou Farahnak, PhD, MD, The surgical clinic, Stockholm South General Hospital

1:5 Report access to necessary resources during the project
Responsible for the safety of the research participants at each recruiting site:

Åsa Dedering, Head of Function Allied Health Professionals, Karolinska University Hospital
Annika Bergqvist, Head of Center for Digestive Diseases, Karolinska University Hospital
Lennart Boström, Head of the surgical clinic, Stockholm South General Hospital

1:6 Applications to additional authorities
NA

2. Information regarding the project

2:1 Summary of the research project
Shorter length of stay in hospitals can be a problem for older people with multiple comorbidities and functional limitations. Therefore, these patients need to be optimally prepared so that they can regain health and functional capacity as soon as possible after a planned hospital stay e.g. undergoing surgery. The aims of the project are to investigate and identify older people at high risk for complications, as well as evaluate if preoperative physical exercise can decrease the risk of complications, mortality, and prolonged length of stay in hospital after abdominal- and thoracic surgery. The observational study will include patients over the age or 70 years scheduled for surgery at Karolinska University Hospital and Stockholm South General Hospital. The association between preoperative physical activity and function and postoperative complications and length of stay will be investigated. The results from this study will form the basis for the inclusion criteria and power calculation for a randomized controlled intervention study were preoperative home-based exercise will be compared to standard preoperative care. Effects on physical function, quality of life, length of stay, complications and mortality will be studied.

2:2 Which/What scientific research questions form the basis of the project?

Observational study
Which factors can predict complications, length of stay in hospital, and mortality after abdominal and thoracic surgery, and identify older people at high risk?

Intervention study
Can preoperative exercise prior to abdominal- and thoracic surgery decrease the risk of functional decline, postoperative complications, and shorten length of stay in older people at high risk?

2:3 Describe results from relevant animal experiments (applicable to clinical treatment research)
NA

2:4 Provide an overview of procedure, data collection and data characteristics

Observational study

The patients' physical activity level, physical function, and health-related quality of life will be assessed preoperatively. Follow-up regarding mortality, length of stay, complications and discharge destination will be conducted at discharge, as well as 30 and 90 days after surgery.

The following data will be collected:

1. Self-reported physical activity measured with the Physical Activity Scale for the Elderly (PASE).
2. Endurance measured with the Six-minute walk test. Number of meters walked during 6 minutes, self-reported effort and dyspnea (Borgs CR-10 scale), oxygen saturation will be recorded.
3. Self-selected and maximal walking speed measured over 10 m, with a 2 meters acceleration- and deceleration phase, as well as lower extremity strength with number of Chair-stands during 30 seconds.
4. Maximal inspiratory and expiratory muscle strength will be measured with MicroRPM.
5. Lung function (FEV_1 and PEF) will be measured with MicroLoop.
6. Grip strength measured with Jamar hand dynamometer.
7. Health-related quality of life will be measured with EORTC QLQ-C30 and QLQ-ELD14. The questionnaire contains 30 questions organised in functional and symptom scales.
8. Cognitive performance will be assessed with Mini Mental State Examination
9. Nutritional status measured with Mini Nutritional Assessment-short form.

The above stated data is not collected as routine data in clinical practice for all patients but will be collected specifically for this study. Background factors such as age, sex, comorbidities, drugs, and smoking habits will be retrieved from the medical records. The research participants will also be tested regarding level of carbon monoxide in exhaled air with a Smokeylyzer.

Mortality, length of stay (at intensive care unit and surgical ward), discharge destination (home/or further care), delirium (with the Confusement Assessment Method), postoperative complications according to the Clavien Dindo classification and patient-reported postoperative symptoms (with the Patient-reported Postoperative Recovery Profile) will be collected. Complications such as infections, wounds, pain, respiratory support (invasive and non-invasive), need of oxygen supplement, and saturation will be documented. Suspected pneumonias will be verified with pulmonary x-ray. For data on mortality the Cause of death registry will be used, additional data will be collected from the medical records except for delirium and self-reported postoperative symptoms, which is not included in routine care. Surgical approach, duration of surgery, and type of postoperative rehabilitation will also be documented. On discharge data collection 1-6 will be repeated (see description above). Data from medical records will be collected after approval from the head of the unit according to laws and regulations.

Statistical analyses

For nominal outcomes such as mortality and discharged destination binominal logistic regression will be used. For postoperative complications according to Clavien-Dindo a multinomial regression analysis will be used with grade 1 as a reference category. Cox regression analysis will be used for analysing length of stay. For continuous outcome variables such as the measures of physical function multiple linear regressions will be used. In the analyses previously described risk factors will be controlled for (age, diabetes mellitus, heart failure, chronic pulmonary disease, systemic disease and smoking, these data are collected from the medical records).

Intervention study

The study is a randomized controlled study where the participants will be randomized to an intervention consisting of preoperative physical exercise or a control group following standard preoperative care. The responsible researcher will perform the randomization. The assessors of physical performance will be blinded to group allocation during the study. This is made possible, as the randomization will be conducted after the baseline assessments. The participants will also receive written information not to reveal their group allocation at follow-up assessments. The intervention period will differ depending on individual variations in preoperative waiting time, but the intervention time will be a minimum of two weeks. To optimize compliance to and quality of the exercise, the intervention will be conducted by a physiotherapist in the participants' home. The results from the observational study will form the basis for possible stratification variables in the intervention study.

Intervention group

The participants will perform individually tailored functional strength training and aerobic training, as well as inspiratory muscle training starting from 30% of maximal inspiratory capacity. On none supervised days the participants will be encouraged to follow recommendations of 30 min physical activity on moderate intensity, and inspiratory muscle training at 20% of maximal capacity. The participants will also receive usual preoperative information.

Control group
The control group will receive usual preoperative information and be encouraged to follow recommendations of 30 min physical activity on moderate intensity at least 5 days/week. Both groups will receive usual postoperative treatment and rehabilitation.

Measurements

At baseline age, sex, comorbidities, smoking habits and living situation will be collected from the medical records. The following measurements will be conducted before the intervention, after the intervention but before surgery, and at discharge from the hospital:

Six-minute walk, gait speed, chair stands, inspiratory muscle strength, lung function, and grip strength. Mortality, length of stay, discharge destination, postoperative complications (pneumonia, stroke, wound infections, pain etc), and comorbidities will be documented after 30 days and 90 days post surgery. Need of oxygen supplement and respiratory support, and saturation will be retained from the medical records. Data from medical records will be collected after approval from the head of the unit according to laws and regulations. Possible complications or adverse events related to testing or training will be documented.

At baseline and 6 month after surgery the participants will fill in three questionnaires (sent by mail) including activities in daily living (ADL-staircase), physical activity level (PASE), and health-related quality of life (EORTC QLQ-C30). All instrument are validated and frequently used in research both nationally and internationally. Reference values from the Swedish population are to be found for the EORTC QLQ-C30.

If the study results in positive findings with decreased length of stay and improved health-related quality of life, calculations regarding health economics will be performed where the costs of preoperative exercise are put in relations to decreased length of stay.

Statistical analyses

Continuous data will be described with mean and standard deviations, and ordinal data with median and interquartile range (q1-q3). Analyses between groups will be performed with intention-to-treat (ITT) analyses and with Repeated measure ANOVA for continuous normally distributed data and Wilcoxon/Kruskal-Wallis test for ordinal data and continuous skewed data. Analyses will be performed in SPPS.

2:5 Report whether collected biological material will be stored in a bio bank
NA

2:6 Documentation, data protection and archiving
Karolinska Institutet, NVS, Division of Physiotherapy will be responsible for that the collected data will be de-identified, coded and stored in a safe way. The Division is also responsible for the code lists and only the PI will have access to these. During the study, at each recruiting site the patients' social security number and study code will be registered in a code key. On the questionnaires and documentation, only the study code will be documented. The participating sites are responsible to handle the collected data de-identified and according to instructions from the PI. After the data collection, material will be stored in a locked safe at Karolinska Institutet only available to the PI and co-applicants of the project. When collecting data from the Cause of death registry, instructions from The National Board of Health and Welfare will be followed.

2:7 Describe previous experiences (own and/or others) of the present procedure, technique or treatment
The PI and co-applicants have extensive experience of collecting data regarding physical activity and function, including pulmonary function. The methods used are well established nationally and internationally. Participating surgeons are responsible for selecting eligible patients so that exclusion criteria are followed and that data are collected without risk for the participants. The intervention could lead to complications such as muscle soreness and joint pain. Muscle soreness is a physiological response to strength training which leads to increased muscle strength. If the participants experience any inconvenience from joints associated with exercise programs or fatigue related to the disease, it will be modified. The randomization process means that half of the included people will not be offered the exercise intervention. Supervised preoperative exercise is not currently offered routinely before surgery and therefore this is considered to be of minor ethical significance. Physical exercise in frail elderly could pose a risk to the individual. However, studies in recent years have shown that this risk is small. The risks of physical inactivity are estimated to be equal or greater. Experiences from using the EORTC QLQ-30 questionnaire to collect data on patients' heath-

related quality of life are positive. The questionnaire is easy to administer and for most people a time frame of less than 10 minutes is needed.

3. Information regarding research participants

3:1 How will the selection of research participants be made?

Observational study

Patients referred to the participating clinics will be informed, orally and in writing, about the study and asked for participation by a research or contact nurse at the first visit in the surgical office.

Inclusion criteria: >70 years of age, understand and speak the Swedish language.

Exclusion criteria: Health status that contradicts physical tests of muscle strength, endurance and functional performance, or a need of acute surgery.

Intervention study

Patients referred to the participating clinics will be consecutively asked for participation in the study by a research or contact nurse at the first visit in the surgical office. For more information see below 3.1 heading Observational study.

Inclusion criteria: >70 years of age, understand and speak the Swedish language. Other inclusion criteria will be based on the results from the observational study.

Exclusion criteria: Health status that contradicts physical tests of muscle strength, endurance and functional performance, or a need of acute surgery within 7 days.

3:2 State the relationship between the researchers/test assessors and the research participants

Therapist (e.g. physician, psychologist, physiotherapist) – research participant (e.g. patient, client)

3:3 Describe the statistical basis for the sample size

For the observational study 200 participants will be recruited, based on experiences from previously published studies with similar research questions.

Power for the intervention study will be calculated based on data from the observational study. A previous study calculated power based on a 10% reduction in postoperative complications. For an 80% (p=.05) power, a total of 584 patients (292 per group) were to be recruited.

3:4 Is it possible that the research participants will be included in several studies at the same time or close to this time period? If so, which type of research?

The study participants will not be included in other exercise studies, however there are several medical research projects (e.g examination of surgical tissue) ongoing in parallel to this study at the participating sites. The research group will make sure that the research participants do not have a higher cumulative risk at participation.

3:5 What insurance cover the research participants?
The patient insurance legislation covers potential adverse events.

3:6 What financial compensation or other benefits are provided to the research persons who participate in the project, and when is it paid?
No patient fee is paid by the participants in the intervention group, otherwise no financial compensation is provided.

4. Information and consent

4:1 The procedure for and the content of the information delivered to the patients at recruitment

Oral information is given in relation to recruitment for the study, if the patient is interested in participating, a written information paper will be provided. Thereafter, the patient will be contacted by the person responsible of recruitment and asked for participation. The information contain information regarding the content and procedure of the study, the research persons are also informed that they can cancel their participation at any time during the study without giving a reason or that it will affect their further care and rehabilitation. Patients who accept participation will be contacted by a physiotherapist to schedule the first assessment.

4:2 How and from who is content collected?
Written informed consent from the research person will be collected at the first assessment by the physiotherapist.

5. Ethical considerations

5:1 Describe all risks anticipated with participation
The physical assessments that will be conducted within the research project are frequently used in clinical practice, well established nationally and internationally, and do not provide any obvious risks for the participants. The choice to perform the exercise in the participants home might be perceived as an intrusion in the private sphere, however it is considered to have a small ethical impact. The value of exercising in the home setting and not having to transport themselves to a clinical is thought to outweigh possible perceptions of an intrusion in the private sphere. In frail older people physical exercise could be a risk for the individual, however previous studies have shown that the risk is small. The risk with physical inactivity is considered to be equal or higher. Close collaboration with physicians at the participating clinics will decrease the risk further.

The risk with physical activity/exercise is not considered higher than the risks of undergoing surgery. Resistance exercise might lead to muscle soreness, however the participant will be informed that muscle sourness related to exercise is an indication of increased muscle activity which in turn is a requirement for increased muscle strength. The exercise and possible adverse events are monitored by the physiotherapist at each supervised session and modified

if needed. The questions regarding quality of life (EORTC QLQ-C30) are used in multiple studies, both in Sweden and internationally. It has also been tested if they are perceived as worrying or offensive, which was not the case.

5:2 Describe the possible benefits for the research persons included in the project
The participants of the study will get an opportunity to a structured assessment of their physical status and function, which is not a part of the standard care and can therefore be considered an advantage giving the participants a full picture of their physical health. Participants randomized to the exercise group will also be given the opportunity to improve their physical function, which can be of importance for their daily activities and postoperative recovery.

5:3 Identify possible ethical problems that might appear from the project
If positive results of the overall project can be shown, the knowledge can benefit older people in general undergoing abdominal- or thoracic surgery. Ethical problems appearing in form of risk in a further perspective is not considered to exist. Even if no positive exercise effects can be shown from the study, the pros of being physically active in general is considered positive in relation to other health factors.

6. Reporting of the results

6:1 How is the principal investigator and participating researchers guaranteed access to data and who is responsible for data processing and report writing?

Only the PI and co-researchers stated in this application will have full access to data. Apart from doctoral students and postdocs involved in the studies, coworkers at participating clinics will be able to assess some data in relation to master thesis and thesis by resident physicians that are under supervision from PI and/or co-applicants of this project.

6:2 How will the results be made publicly available? Will the study be submitted for publication in a journal or be published in any other way?

The results will be published in doctoral thesis, scientific journals and in scientific essays on advanced level such as master, residency etc, as well as in popular science contexts directed towards health professionals and the public.

6:3 In what way will the research participants be guaranteed integrity when the material is made public/is published?

During the data collection all participants are de-identified, and the results will only be presented on a group level.

7. Economical interests and interdependencies

7:1 If commissioned research
NA

7:2 Describe possible economical agreements with public principals or other financiers
NA

7:3 Describe own interests of the entity principally responsible for the research, the principal researcher and the co-applicants
NA

8. Signatures

DECISION: Dnr 2015/1179-31 APPROVED 2015-09-02

Amendment to previously approved ethical application Dnr.2015/1179-31

The amendment consists of four parts: 1, Validation of the instrument Patient-specific function scale; 2, Revision of previously stated exercise program related to new findings in the research area; 3, A complementary intensified nutrition intervention; 4. Interview study

1.

The instrument Patient-specific function scale has been translated into Swedish and evaluated on a Swedish study population undergoing hand surgery. The purpose of this study is to evaluate the instrument's content and construct validity in people over 70 years of age undergoing abdominal surgery. The study will be carried out within a feasibility study conducted before the previously approved intervention study, also described below. The research participants will complete the form together with the physiotherapist responsible at the first and last visit (see below). Other measurement methods to be used in the study are stated in the previously approved application.

2.

The previously approved research plan stated the following:

> a) *”Participants will train inspiratory muscle strength where they will breathe against a resistance at 30% of their maximum capacity to start with. The training should be perceived as strenuous (5 on Borg's CR scale) and the resistance is increased/decreased in relation to this. The training will be performed 2-3 times a week for 15 minutes.”*

As new findings indicate that the training should be of high-intensity but with shorter intervals, the participants will now train at 50-70% of their maximum capacity and perform 30 breaths x2 at this level. The training will take place daily, but under the supervision of the physiotherapist 2-3 days/w.

> *b) ”Individually adapted functional training.... This will be combined with functional exercises related to each individual's needs, such as to carry and move things. ”*

In addition the participants will, together with the physiotherapist, complete the form "Patient-specific function scale" where the participant can describe three important activities that they have problems performing or cannot perform at all. They then will estimate how difficult they think it is to perform the activity on a scale from 0=cannot perform the activity to 10=can perform the activity without any problem. These activities will be incorporated in the training program and followed up after the intervention. See Appendix 1 for updated patient information.

3.

Purpose

To evaluate whether a combination of pre-operative physical activity and an intensified nutritional treatment prior to abdominal surgery increases appetite and nutritional intake and thus prevents the reduction of muscle mass and physical function in older patients at risk of malnutrition.

Overview of topic

The number of frail older people is increasing in line with an aging population. Protein requirements increase with increasing age, while disease and treatment can lead to several inhibiting factors for appetite and nutritional intake. Malnutrition can therefore be a problem

before and after treatment has been completed. Loss of muscle mass and function can be directly harmful and can be associated with poorer prognosis. Strength training can improve muscle function and balance in frail older people, but on the other hand can put a patient in a catabolic state unless energy and protein are supplied in sufficient amount. Computer tomography, CT is generally used to identify the presence of tumor but not in the evaluation of malnutrition.

Method
Design; Intervention study
Data source; The study is a randomized controlled trial in which the participants are randomized to an intervention consisting of preoperative exercise or control group consisting of standard treatment. **Supplement:** Patients screened for risk of malnutrition in both study arms will be offered an intensified nutrition treatment by a dietician. According to a power calculation based on effects on nutrient variables, 40 patients/arm are required in the RCT study.
Method; Screening of the risk of malnutrition is conducted with three questions according to recommendations from the Swedish Association of Local Authorities and Regions: involuntary weight loss; eating disorders; and BMI.
The patients who are at risk of malnutrition are asked to complete a three-day diet diary and be scheduled for a dietician contact at; 1) the first visit 2) time of surgery 3) 8 weeks after discharge, and 4) 1 year after surgery. In addition, telephone follow-up/support will be provided every week until surgery. Intensified nutrition treatment by a dietician involves an investigation of dietary intake and individually adapted dietary advice with a focus on energy and protein-rich foods and drinks as well as meal arrangements. If necessary, protein-rich diet supplements are prescribed.
To evaluate energy and protein intake, the program DietistNet will be used. At the same time, this gives us the opportunity to study adherence to the given dietary treatment and the amount of dietary supplements consumed.
Standard CT images should be analyzed by a dietician to determine body composition and changes in body composition, during first visit and at 1-year follow-up. DT imagining are already a part of standard care and require no extra examination of the patient. See Appendix 1 for updated patient information.

Significance: Few studies have investigated how diet and nutritional intake can be affected by physical activity in frail older people, although we know that malnutrition is a problem in health care. Adherence to prescribed diet and diet supplements is rarely studied. CT is taken to determine diagnosis and to follow and evaluate treatment. With the result of the measurements, we can determine the degree of muscle loss and observe specific tissue changes without any additional inconvenience to the patient or cost to healthcare.
If this supplement in the study shows a positive effect, this additional application can lead to increased collaboration between dieticians and physiotherapists in both open and inpatient care with a strengthened care chain, improved care and, above all, improved care for frail older patients undergoing advanced surgery.

Ethical considerations: The original study is approved by the Regional Ethics Board in Stockholm (Dnr 2015/1179-31), but an additional application is needed for the specific nutrition intervention. Study patients will receive oral and written information in accordance with the original study and informed consent will be obtained before inclusion. Selected methods and measuring instruments are validated and used in similar studies in other patient groups.

4.

Overview of topic

Previous studies indicate that optimization of physical function and nutritional status before surgery can reduce the risk of complications. Patients would like to have their surgery completed as soon as possible and the standardized care processes that are now being introduced in Sweden within cancer care decreases the time for optimization. Until now, there are no medical indications that a waiting period of 3-4 weeks instead of 2-3 weeks implies poorer cancer-related outcomes.

Purpose

The study aims to describe patients' view of postponing the operation a couple of weeks in order to be able to improve their physical function and nutritional status prior to surgery.

Method

Approximately 20-30 patients who meet the criteria for inclusion in the on-going observational study will be asked about participation (Dnr 2015/1179-31). Oral and written information are provided by the research nurse, and written consent is obtained from the physiotherapist. Both those who are included in and declines participation in the observation will be approached. The interviews will take place before the surgery. All interviews will be recorded with a dictaphone. Recorded interviews will be transcribed and analyzed using an appropriate method. A code list of participants will be created and the list will be stored separate from the voice recordings and transcripts. Only the study leader has access to the code list.

Ethical considerations

It is important to emphasize that the research persons' participation in the study is voluntary and that they can cancel their participation at any time during the interview. The interview questions could be perceived as an intrusion into the private sphere, but this can be weighed against the fact that the research persons has the opportunity to express themselves and describe their situation by participating in the project. Data is presented without possibility of individual identification of research subjects.

Huddinge 10th of August 2018

DECISION: Dnr 2016/1587-32 APPROVED 2016-08-26
