# Supplementary material for: Feasibility of preoperative supervised home-based exercise in older adults undergoing colorectal cancer surgery – A randomized controlled design
Source: PLoS One. 2019 Jul 2;14(7):e0219158. doi: 10.1371/journal.pone.0219158 (PMC6605852; doi:10.1371/journal.pone.0219158)
Supplement: S2 File — (PDF) [file pone.0219158.s003.pdf]

Beslutad 2013-03-04

ANSÖKAN OM ETIKPRÖVNING AV FORSKNING SOM AVSER MÄNNISKOR

Information till ansökan, ***se Vägledning till ansökan*** **(*www.epn.se*)**
Beroende på vilken forskning som ansökan gäller kommer de uppgifter som efterfrågas att ha olika relevans. Vid ändring av tidigare godkänd ansökan, se ***Vägledning till ansökan.***

Till Regionala etikprövningsnämnden i: Stockholm

Den regionala etikprövningsnämnd till vars upptagningsområde forskningshuvudmannen hör, se respektive nämnd *(www.epn.se)*.

Avgift inbetald datum:

Observera att en ansökan aldrig är komplett och därmed kan behandlas förrän blanketten är korrekt ifylld och avgiften är betald.

Projekttitel: Better in Better out: Preoperativ träning för att minska risken för komplikationer och ge kortare vårdtider för sköra äldre efter buk- och thoraxoperation

Ange en beskrivande titel på svenska för lekmän. Titeln ska ej innehålla sekretesskyddad information. Ange också i förekommande fall, t.ex. vid klinisk läkemedelsprövning, projektets identitet, forskningsplanens/protokollets nummer, version, datum. Vid ändring av tidigare godkänd ansökan, se Vägledning till ansökan.

Projektnummer/identitet: Version nummer: EudraCT nr (vid läkemedelsprövning):

Uppgifter som fylls i av den regionala etikprövningsnämnden

Ansökan komplett:	Dnr:
Begäran om ytterligare information (i sak):	Begärd information inkommen:
Beslutsdatum:	Expeditionsdatum:

Ansökan avser (gäller även vid begäran om rådgivande yttrande):

Forskning där endast en forskningshuvudman deltar (5 000 kr)	☒
Forskning där mer än en huvudman deltar (16 000 kr)	☐
Forskning där mer än en forskningshuvudman deltar, men där samtliga forskningspersoner eller forskningsobjekt har ett omedelbart samband med endast en av forskningshuvudmännen (5 000 kr)	☐

Endast behandling av personuppgifter (5 000 kr) ☐
(När enbart redan befintliga personregister ska användas, t. ex. nationella databaser)

Forskning som gäller klinisk läkemedelsprövning (16 000 kr) ☐

Ändring av tidigare godkänd ansökan enligt 4 § förordning (2003:615) om etikprövning av forskning som avser människor (2 000 kr) ☐

Om nämnden finner att forskningsprojektet inte faller inom etikprövninglagens tillämpningsområde önskas ett rådgivande yttrande. (Se 4a och 4b §§ i förordning 2003:615 och Vägledning till ansökan)
Ja: ☒ Nej: ☐

1. Information om forskningshuvudman m.m.

1:1 Forskningshuvudman (Se p. 1:1 i Vägledning till ansökan)

Ansökan om etikprövning av forskning ska göras av forskningshuvudmannen. *Med forskningshuvudman avses en statlig myndighet eller en fysisk eller juridisk person i vars verksamhet forskningen utförs.*

Namn: Karolinska Institutet, Inst för neurobiologi, vårdvetenskap och samhälle
Adress: Alfred Nobels Allé 23, D3, 14183 Huddinge

1:2 Behörig företrädare för forskningshuvudmannen

Behörig företrädare är t.ex. prefekt, enhetschef, verksamhetschef. Forskningshuvudmännen bestämmer själva, genom interna arbets- och delegationsordningar eller genom fullmakt, vem som är behörig att företräda forskningshuvudmannen.

Namn: Maria Eriksdotter Tjänstetitel: Prefekt
Adress: Karolinska Institutet, Inst för neurobiologi, vårdvetenskap och samhälle, Alfred Nobels Allé 23, D3, 14183 Huddinge

1:3 Forskare som är huvudansvarig för genomförandet av projektet (kontaktperson)

(Se p. 9 bil. nr 10 och p. 1:3 i Vägledning till ansökan)

Observera! Den som är huvudansvarig forskare ansvarar för att andra medverkande som ska genomföra projektet har tillräcklig kompetens (vetenskaplig och klinisk) och vid läkemedelsprövning har tillräcklig kunskap om "Good Clinical Practice" (GCP). Vid doktorandstudier är som regel handledaren huvudansvarig forskare.

Namn: Elisabeth Rydwik Tjänstetitel: Adjunkt, Docent, leg sjukgymnast
Postadress: Karolinska Institutet, Inst. för NVS, Sektionen för fysioterapi, Alfred Nobels Allé 23, 23100, 141 83 Huddinge
E-postadress: elisabeth.rydwik@ki.se
Telefon:
Mobiltelefon: 0736-209783

1:4 Andra medverkande (Se p. 9 bil. nr 1 och p. 1:4 i Vägledning till ansökan)

Övriga deltagande forskningshuvudmän samt forskare ansvariga för att lokalt genomföra projektet (kontaktpersoner) anges här eller i bilaga med namn och adresser (se p. 9 bil. nr 1).

Erika Franzén, docent, leg sjukgymnast. Karolinska Universitetssjukhuset, Fysioterapikliniken; Karolinska Institutet, Inst för Neurobiologi, Vårdvetenskap och Samhälle, Sektionen för Fysioterapi

Åsa Dedering, docent, leg sjukgymnast. Verksamhetschef för Fysioterapikliniken, Karolinska Universitetssjukhuset

Malin Nygren-Bonnier, med dr, leg sjukgymnast. Karolinska Universitetssjukhuset, Fysioterapikliniken; Karolinska Institutet, Inst för Neurobiologi, Vårdvetenskap och Samhälle, Sektionen för Fysioterapi

Mia Bergenmar, docent, universitetssjuksköterska, Gastrocentrum, Karolinska Universitetssjukhuset, Ins för Onkologi-Patologi, Karolinska Institutet

Bengt Isaksson, docent, överläkare, Gastrocentrum, Karolinska Universitetsjukuhuset

Monika Egenvall, med dr, överläkare, Gastrocentrum, Karolinska Universitetsjukuhuset

Parastou Farahnak, med dr, biträdande överläkare, Kirurgkliniken, Södersjukhuset

1:5 Redovisa tillgång till nödvändiga resurser under projektets genomförande

(Se p. 9 bil. nr 9 och p. 1:5 i Vägledning till ansökan)

Ange vem/vilka som har ansvaret (prefekt, verksamhetschef eller motsvarande) för forskningspersonernas säkerhet vid alla enheter/kliniker där forskningspersoner ska delta. Intyg från dessa ansvariga *ska* bifogas (se p. 9 bil. nr 9). Av intyget ska framgå att erforderliga ekonomiska, strukturella och personella resurser finns tillgängliga för att garantera forskningspersonernas säkerhet.

Åsa Dedering Stephansson, verksamhetschef, Fysioterapikliniken, Karolinska Universitetssjukhuset

Annika Bergqvist, verksamhetschef, Gastrocentrum, Karolinska Universitetssjukhuset

Lennart Boström, verksamhetschef, vo Kirurgi, Södersjukhuset

1:6 Ansökan/anmälan till andra myndigheter i vissa fall

(se p. 1:6 i Vägledning till ansökan)	**Insänd**	**Datum**
a)Vid klinisk läkemedelsprövning: Läkemedelsverket	☐	

b)Vid inrättande av biobank: Socialstyrelsen ☐

c)Vid undersökning omfattande joniserande strålning: Strålskyddskommitté ☐

2. Uppgifter om projektet

2:1 Sammanfattande beskrivning av forskningsprojektet

(Se p. 9 bil. nr 2 och p. 2:1 i Vägledning till ansökan)

Beskrivningen ska kunna förstås av nämndens samtliga ledamöter. Undvik därför terminologi som kräver specialkunskaper. Ange bakgrund och syfte för studien samt den/de vetenskapliga frågeställning (ar) som man söker svar på. Ange de viktigaste undersökningsvariablerna. Beskriv vilka kunskapsvinster projektet kan förväntas ge och betydelsen av dessa. Ange om det är en registerstudie, uppdragsforskning etc. För fackmän avsedd detaljerad information om forskningsplan/protokoll (program) *ska* bifogas som bilaga (se p. 9 bil. nr 2). För utformning av forskningsplan/protokoll se p. 2:1 i Vägledning till ansökan. Ange när datainsamlingen beräknas vara avslutad. En utförligare beskrivning av studiens genomförande *avsedd för lekmän* kan vid behov bifogas den för fackmän avsedda obligatoriska forskningsplanen.

Korta vårdtider kan vara ett problem för äldre personer med flera samtidiga sjukdomar och funktionsnedsättningar. Patienterna behöver därför förberedas på ett optimalt sätt så att de återfår hälsa och funktion så snabbt som möjligt efter t.ex. en operation. Syftet med studien är att kartlägga och identifiera äldre personer med hög risk för komplikationer samt att sedan undersöka om preoperativ träning minskar risken för komplikationer och mortalitet samt förkortar vårdtiderna efter buk- och thoraxoperation. Kartläggningsstudien kommer att inkludera patienter över 70 år som planeras för operation vid Karolinska Universitetssjukhuset och Södersjukhuset. Sambandet mellan fysisk aktivitet och funktion innan operation och mortalitet, komplikationer och vårdtid efter operation kommer att undersökas. Resultat från denna studie kommer att ligga till grund för inklusionskriterier och powerberäkning för en randomiserad kontrollerad interventionsstudie där preoperativ träning i hemmet kommer att jämföras med sedvanlig preoperativ behandling. Effekter på funktionsförmåga, livskvalitet, vårdtid, komplikationer samt mortalitet kommer att studeras.

2:2 Vilken/vilka vetenskaplig (a) frågeställning (ar) ligger till grund för projektets utformning?

Om projektet kan karakteriseras som en hypotesprövning, ange den primära och eventuellt sekundära hypotesen. Hänvisning till mer detaljerad information för fackmän kan ske till bifogad forskningsplan/protokoll enligt punkt 2:1.

Kartläggningsstudie

Vilka faktorer kan predicera komplikationer, vårdtider och mortalitet efter buk- och thoraxoperationer och identifiera äldre personer med hög risk att drabbas av detta?

Interventionsstudie

Kan preoperativ träning inför buk- och thoraxoperation minska risken för funktionsnedsättning, komplikationer och mortalitet samt ge kortare vårdtider hos äldre personer med hög risk?

2:3 Redogör för resultat från relevanta djurförsök (Gäller klinisk behandlingsforskning)

Om djurförsök inte utförts ange skälen till detta.

NA

2:4 Redogör översiktligt för undersökningsprocedur, datainsamling och datas karaktär

(Se p. 9 bil. nr 5 och p. 2:4 i Vägledning till ansökan)

Av beskrivningen ska framgå hur projektet planeras genomföras. Beskriv insamlade datas karaktär. Ange hur datas tillförlitlighet säkerställs (t.ex. kvalitetskontroll/monitorering). Vid enkäter och intervjuer ska beskrivas tillvägagångssätt och t.ex. frågors innehåll och hur slutsatser dras. Enkäter och skattningsskalor *ska* bifogas (se p. 9 bil. nr 5). För medicinsk forskning ska anges t.ex. typer av ingrepp, mätmetoder, antal besök, tidsåtgång vid varje försök, doser och administrationssätt för eventuella läkemedel och/eller isotoper, blodprovsmängd (även ackumulerad mängd vid multipla försök). Ange om och på vilket sätt undersökningsprocedur m.m. skiljer sig från klinisk rutin. Om en behandling studeras för första gången på människa ska detta framgå och relevanta säkerhetsrutiner beskrivas. Ange proceduren för att ge den eventuella behandling efter projektets slut, som kan erfordras. Ange procedur för insamling av biologiskt material. Redogör för datakällor och procedurer vid behandling av personuppgifter. För mer detaljerad information kan hänvisning ske till bilagd forskningsplan.

Observationsstudie

Patienternas fysiska aktivitetsnivå, funktion och hälsorelaterade livskvalitet undersöks innan operation. Uppföljning avseende mortalitet, vårdtider, komplikationer och utskrivningsdestination görs vid utskrivning samt 30 och 90 dagar efter operation.

Följande data kommer att samlas in:

1. Självskattad fysisk aktivitet mäts med Physical Activity for the Elderly scale (PASE, se bilaga 5A).

2. Uthållighet mäts med six-minute walk, där personen går fram och tillbaka i självvald gånghastighet under 6 minuter och där max antal meter samt självskattad ansträngning och andfåddhet (Borgs CR-10 skala), samt saturation innan och direkt efter noteras.

3. Självvald och maximal gånghastighet över 10 m, med 2 meters accelerations- och decclerationsfas samt antal uppresningar till stående (chair-stand) under 30 sekunder kommer att mätas.

4. Maximal inspiratorisk muskelstyrka och uthållighet mäts med MicroRPM.

5. Lungfunktion (FEV1 och PEF) mäts med MicroLoop.

6. Handstyrka mäts med Jamar handdynamometer

7. Hälsorelaterad livskvalitet mäts med EORTC QLQ-C30 och EORTC QLQ-ELD14. Det består av 30 frågor som är organiserade i funktionsskalor och symptomskalor (se beskrivning nedan samt bilaga 5B).

8. Kognitiv funktion mäts med Mini Mental State Examination.

9. Nutritionstillstånd mäts med Mini Nutritional Assessment-short form.

Ovanstående data samlas inte in i klinisk rutin för alla patienter utan kommer att samlas in specifikt för studien. Bakgrundsfaktorer som ålder, kön, co-morbiditet, läkemedel, rökvanor hämtas från journaldokumentationen. Forskningspersonerna kommer också att testas med avseende på grad av kolmonoxid i utandningsluften med Smokeylyzer.

Mortalitet, vårdtid (intensivvård respektive vårdavdelning), utskrivningsdestination (hemmet/annan vårdnivå), delirium (skalan Confusement Assessment Method, se bilaga 5C), postoperativa omplikationer enligt Clavien-Dindo och självrapporterade postoperativa symtom (Patient-reported Postoperative Recovery, se bilaga 5D) hämtas in. Komplikationer såsom infektion, sår, smärta, andningsstöd (invasiv och non-invasiv ventilation) behov av extra syrgas, blodgaser och saturation kommer att dokumenteras. Vid misstanke om lunginflammation kommer den att verifieras med lungröntgen. För uppgifter om mortalitet används data från Dödsorsaksregistret, övriga data hämtas från journaldokumentationen förutom bedömning av delirium samt självrapporterade postoperativa symtom som inte undersöks i klinisk rutin. Typ av operation, operationstid (total och ingreppstid) och typ av postoperativ rehabilitering kommer också att dokumenteras. Inför utskrivning upprepas datainsamling 1-6 (se beskrivning ovan). Data som inhämtas ur den medicinska journalen sker efter tillstånd av verksamhetschefen enligt gällande lagar och förordningar.

Statistisk analys

För nominala utfallsmått som mortalitet och utskrivningsdestination kommer binominal logistisk regression att användas. För postoperativa lungkomplikationer enligt Clavien-Dindo kommer multinominal regressionsanalys att användas där grad 1 används som referenskategori. För vårdtid kommer Cox regressionsanalys att användas. I de multipla regressionsanalyserna kommer sedvanliga tidigare beskrivna riskfaktorer att kontrolleras för (ålder, diabetes mellitus, hjärtsvikt,

kronisk lungsjukdom, systemiska sjukdomar och rökning, dessa data inhämtas från journaldokumentationen). Alla analyser kommer att utföras i SPSS.

Interventionsstudie

Studien är en randomiserad kontrollerad studie där deltagarna randomiseras till intervention bestående av preoperativ träning eller kontrollgrupp bestående av sedvanlig behandling. Randomiseringsprocessen utförs av ansvarig huvudforskare. Utvärderararna kommer att vara blindad för deltagarnas grupptillhörighet. Det kommer att möjliggöras genom att randomiseringen sker efter genomförda baslingemätningar, samt att deltagarna skriftligen uppmanas att inte delge grupptillhörighet vid uppföjlning. Beroende på beslut om operationstid så kommer interventionsperioden att variera. För avsedd effekt bör dock interventionsperioden vara minst två veckor. För att möjliggöra följsamhet till och optimal kvalitet av träningsprogrammet genomförs det under ledning av fysioterapeut i patientens hem. Randomiseringen sker konsekutivt under ledning av ansvarig forskare. Resultatet i observationsstudien kommer att ligga till grund för eventuella stratifieringsvariabler i interventionsstudien.

Interventionsgrupp

Deltagarna kommer att träna individanpassad funktionell styrketräning och uthållighetsträning, samt träna inspiratorisk muskelstyrka där de får andas mot motstånd och börja med 30% av deras maximala kapacitet. På icke träningsledda dagar uppmanas deltagarna att följa rekommendationerna om 30 min måttlig ansträngande fysisk aktivitet samt träna inspiratorisk muskelträning på 20% av deras maximala kapacitet. Inför operation får deltagarna sedvanlig preoperativ information.

Kontrollgrupp

Deltagarna får sedvanlig preoperativ information men uppmanas också att följa rekommendationerna om 30 min måttlig ansträngande fysisk aktivitet minst 5 dgr/v.

Deltagarna i båda grupperna får sedvanlig postoperativ behandling och rehabilitering.

Mätmetoder

Mätningarna sker innan intervention, efter avslutad intervention dagen innan operation, samt dagen innan utskrivning till rehabilitering/hemmet.

Följande mätmetoder används i interventionsstudien:

Vid baslinjen kommer ålder, kön, co-morbiditet, läkemedel, rökvanor samt boendeförhållanden att inhämtas från journaldokumentationen.

Six-minute walk, gånghastighet, antal uppresningar till stående, inspiratorisk maximal och uthållighetsmuskelstyrka, lungfunktion och handstyrka mäts innan intervention, innan operation samt vid utskrivning efter operation. Mortalitet, vårdtid, utskrivningsdestination, postoperativa komplikationer (lunginflammarion, stroke, sår, infektioner, smärta etc) och co-morbiditet dokumenteras vid utskrivning samt efter 30 och 90 dagar. Behov av syrgas och andningsstöd samt resultat av blodgastester och sauturation hämtas ifrån den medicinska journalen. Data som inhämtas ur den medicinska journalen sker efter tillstånd av verksamhetschefen enligt de lagar och förordningar som gäller. Eventuella komplikationer och biverkningar i samband med test och träning dokumenteras.

Vid baslinjen och sex månader efter operation erhåller deltagarna tre frågeformulär per post om grad av oberoende i dagliga aktiviteter (ADL-trappan, se bilaga 5E), fysisk aktivitetsnivå (PASE) och hälsorelaterad livskvalitet (EORTC QLQ-C30). Alla instrumenten är validerade och testade i ett flertal studier både internationellt och i Sverige. För EORTC QLQ-C30 finns normdata för den svenska befolkningen.

Om studien visar på positiva signifikanta effekter på kortare vårdtider eller hälsorelaterad livskvalitet, kommer hälsoekonomiska beräkningar att göras, där kostnader för preoperativ träning sätts i relation till grad av förkortade vårdtider.

Statistisk analys

Kontinuerliga data kommer att beskrivas med medelvärde och standardavvikelse och ordinala data med median och interquartile range (q1-q3). Analyser mellan grupper kommer att utföras med intention-to-treat (ITT) analys med Repeated measure ANCOVA för kontinuerliga normalfördelade data och Wilcoxon/Kruskal-Wallis test för ordinal data och kontinuerliga snedfördelade data. Alla analyser kommer att utföras i SPSS.

2:5 Redogör för om insamlat biologiskt material kommer att förvaras i en biobank

(Se p. 2:5 i Vägledning till ansökan)

Med biobank avses biologiskt material från en eller flera människor som samlas och bevaras tills vidare eller för en bestämd tid och vars ursprung kan härledas till den eller de människor från vilka materialet härrör. Redogör för var och hur prover som ska sparas förvaras, kodningsprocedurer och villkor för utlämnande av prover. Ange huvudman för biobanken.

NA

2:6 Dokumentation, dataskydd och arkivering (Se p. 2:6 i Vägledning till ansökan)

Redogör för hur undersökningsprocedurer och eventuella ingrepp dokumenteras. Ange om band- och videoinspelningar används. Om materialet ska kodas, ange proceduren, vem som förvarar kodlistor/kodnycklar och vem eller vilka som har tillgång till dem, var och hur länge de förvaras samt om materialet kommer att anonymiseras eller förstöras. Redogör för vilken tillgänglighet datamaterialet har och hur det förvaras samt hur erforderligt sekretesskydd erhålls.

Karolinska Institutet, NVS, Sektionen för fysioterapi kommer att ansvara för att insamlade data avkodas och förvaras inlåsta och avidentifierade. Enheten ansvarar också för kodlistor och endast huvudansvarig forskare kommer att ha tillgång till dessa. På varje klinik upprättas en kodlista där varje patients personnummer och det kodnummer patienten får registreras. På frågeformulären och dokumentationshandlingarna anges endast kodnumret. Medverkande kliniker ansvarar för att insamlade data hanteras och avkodas enligt instruktion från huvudansvarig forskare. Material förvaras inlåsta på Karolinska Institutet och endast huvudansvarig forskare samt övriga medsökande forskare i projektet har tillgång till dem. Vid inhämtning av registerdata från Dödsorsaksregistret kommer Socialstyrelsen direktiv att följas.

2:7 Redogör för tidigare erfarenheter (egna och/eller andras) av den använda proceduren, tekniken eller behandlingen

Särskilt angeläget är att redovisning av risker för komplikationer görs tydliga och i förekommande fall med angivande av relevanta publikationer. Vid nya behandlingar av patienter, t.ex. med läkemedel, bör anges hur många patienter (med aktuell eller annan åkomma) som tidigare erhållit föreslagen behandling, läkemedelsdosering (eller annan dosering) samt hur långa behandlingsperioder som studerats.

Huvudansvarig forskare och flera av medsökande har stor erfarenhet av att samla in data kring fysisk aktivitet och fysisk funktion inklusive lungfunktion. Metoderna är väl etablerade både nationellt och internationellt. Medsökande överläkare ansvarar för att exklusionskriterier efterföljs och att medicinska data insamlas utan risker för forskningspersonerna. Interventionen skulle kunna leda till komplikationer såsom träningsvärk, ledbesvär och trötthet. Träningsvärk är en positiv effekt av styrketräning vilket leder till ökad muskelstyrka. Om forskningspersonerna upplever eventuella besvär från leder samt trötthet i samband med träningsprogrammen kommer dessa att modifieras. Randomiseringsprocessen innebär att hälften av de inkluderade personerna inte kommer att erbjudas intervention i form av träning. Träning erbjuds inte i nuläget inför operation och därför anses detta ha en ringa etisk betydelse. Erfarenheterna är goda av att använda EORTC QLQ-30 för att samla in data om patienters hälsorelaterade livskvalitet. Frågeformuläret tycks varken vara upprörande eller svårt att fylla i och för de allra flesta tar det mindre än 10 minuter att fylla i.

3. Uppgifter om forskningspersoner

3:1 Hur görs urvalet av forskningspersoner? (Se p. 9 bil. nr 3 och p. 3:1 i Vägledning till ansökan)

Med forskningsperson avses en levande människa som forskningen avser. Ange urvalskriterier (inklusion och exklusion). Redogör för på vilket sätt forskaren kommer i kontakt med/får kännedom om lämpliga forskningspersoner. Om annonsering sker, *ska* annonsmaterialet insändas som bilaga (se p. 9 bil. nr 3). Om t.ex. barn eller personer som tillfälligt eller permanent inte är kapabla att ge ett eget informerat samtycke ska ingå i projektet, ska detta särskilt motiveras. Om vissa grupper utesluts från deltagande i projektet ska detta särskilt motiveras.

Observationsstudie

Patienter som remitteras till medverkande kliniker informeras muntligt och skriftligt om studien och tillfrågas om deltagande av ansvarig forskningssjuksköterska/kontaktsjuksköterska vid nybesöket.

Inklusionkriterier: >70 år, förstår och pratar svenska.

Exklusionkriterier: Hälso- och funktionstillstånd som förhindrar test av muskelstyrka, uthållighet och funktionsförmåga samt behov av akut operation.

Interventionsstudie

Berörda patienter vid medverkande kliniker kommer konsekutivt tillfrågas om deltagande i studien av forskningssjuksköterska/kontaktsjuksköterska i samband med nybesök. I övrigt se ovan under 3.1, rubrik Observationsstudie.

Inklusionskriterier: >70 år, förstår och pratar svenska. Övriga inklusionskriterier kommer att beslutas utifrån resultatet i kartläggningsstudien.

Exklusionkriterier: Hälso- och funktionstillstånd som förhindrar test av muskelstyrka, uthållighet och funktionsförmåga samt deltagande i träning och akut behov av operation inom 7 dagar.

3:2 Ange relationen mellan forskare/försöksledare och forskningspersonerna

- [x] Behandlare (t.ex. läkare, psykolog, sjukgymnast) - forskningsperson (t.ex. patient, klient)
- [ ] Kursgivare (lärare) - student
- [ ] Arbetsgivare - anställd
- [ ] Annan relation som kan tänkas medföra risk för påverkan. Beskriv:

3:3 Redogör för det statistiska underlaget för studiepopulationens (-ernas)/ undersökningsmaterialets (-ens) storlek (Se p. 3:3 i Vägledning till ansökan)

Redovisa statistisk styrka, så kallad "power"- beräkning eller redovisa motsvarande överväganden som tydliggör studiens möjligheter att besvara frågeställningarna.

Till observationsstudien kommer 200 personer rekryteras. Detta baseras på erfarenheter från andra publicerade studier med liknande frågeställning.

Power för interventionsstudien kommer att beräknas utifrån data i kartläggningsstudien. En tidigare studie beräknade power, utifrån att 10% minskning av postoperativa komplikationer ansågs vara kliniskt relevant, och 584 patienter (292 i varje grupp) beräknades då behöva inkluderas med en power på 80% (p=.05)

3:4 Kan forskningspersonerna komma att inkluderas i flera studier samtidigt eller i nära anslutning till denna? I så fall, vilken typ av forskning?

(Se p. 3:4 i Vägledning till ansökan)

Forskningspersonerna kommer inte att ingå i några andra träningsstudier, men det pågår flera medicinska forskningsprojekt (t.ex. undersökning av bortopererat preparat) parallellt på medverkande kliniker. Forskargruppen kommer att säkerställa att forskningspersonerna sammantaget inte löper ökad risk vid deltagande.

3:5 Vilket försäkringsskydd finns för de forskningspersoner som deltar i projektet?

Det åligger forskningshuvudmannen att kontrollera att det finns försäkring som täcker eventuella skador som kan uppkomma i samband med forskningen.

Patientförsäkringen täcker eventuella skador som kan uppkomma.

3:6 Vilken ekonomisk ersättning eller andra förmåner utgår till de forskningspersoner som deltar i projektet och när betalas ersättningen ut? Utförligare beskrivning kan lämnas i bilaga.

(Se p. 9 bil. nr 11 och p. 3:6 i Vägledning till ansökan)

Ersättning för obehag och besvär. Ange belopp (före skatt):

Ersättning för förlorad arbetsinkomst	☐ Ja	☒ Nej
Reseersättning	☐ Ja	☐ Nej
Befrielse från kostnader för läkemedel	☐ Ja	☒ Nej

Befrielse från andra kostnader. Vilka? Patientavgift för deltagare i interventionsgruppen

Andra förmåner. Vilka?

När betalas ersättningen ut?

Ingen ersättning betalas ut ☒

4. Information och samtycke

4:1 Proceduren för och innehållet i den *information* som lämnas då forskningspersoner tillfrågas om deltagande

(Se p. 9 bil. nr 4 och Vägledning till forskningspersonsinformation.)

Enligt 16 § lag (2003:460) om etikprövning av forskning som avser människor ska forskningspersonen informeras om den övergripande planen för forskningen, syftet med forskningen, de metoder som kommer att användas, de följder och risker som forskningen kan medföra, vem som är forskningshuvudman, att deltagande i

forskningen är frivilligt och forskningspersonernas rätt att när som helst avbryta sin medverkan. Beskriv hur och när information ges och vad den innehåller. Ange vem som informerar. Normalt ska en kortfattad och lättförståelig skriftlig information ges. Denna skriftliga information *ska* bifogas ansökan (se p. 9 bil. nr 4). Om ingen eller ofullständig information ges, måste skälen för detta noggrant anges.

Muntlig information sker i samband med rekrytering till studien vid förfrågan om deltagande, om forskningspersonen är intresserad av att delta lämnas en skriftlig information ut. Personen blir sedan kontaktad av rekryteringsansvarig och tillfrågad om deltagande. Informationen innehåller information om studiens innehåll och genomförande, forskningspersonen informeras också om att han/hon kan avbryta studien när som helst utan att ange orsak och utan att det påverkar framtida vård och rehabilitering (se bilaga 4 a, b). De som tackar ja blir kontaktade per telefon av ansvarig fysioterapuet för att säkerställa deltagande samt bli kallade till undersökning.

4:2 Hur och från vem inhämtas *samtycke*? (Se Vägledning till forskningspersonsinformation)

Beskriv proceduren; vem som frågar, när detta sker och hur samtycket dokumenteras. Utförlig redovisning är särskilt viktig då barn eller personer med nedsatt beslutskompetens ingår i studien, likaså vid studier av en grupp/grupper, t.ex. skolklasser, föreningar, organisationer, företag, kyrkosamfund, församlingar eller grupper som interagerar inom sociala medier.

Frågan om samtycke inhämtas skriftligen från forskningspersonen av fysioterapeuten i samband med första undersökningstillfället (se bilaga 4 c, d).

5. Forskningsetiska överväganden

5:1 Redogör för alla risker som deltagandet kan medföra

Dessa kan vara t.ex. fysisk eller psykisk skada, smärta, obehag eller integritetsintrång på kort eller lång sikt. Ange vilka åtgärder som har vidtagits för att förebygga riskerna som nämns ovan samt vilken beredskap som finns för att hantera sådana komplikationer. Ange vilka/de metoder som kommer att användas för att efterforska, registrera och rapportera oönskade händelser.

De fysiska undersökningar som kommer att genomföras inom ramen för forskningsprojektet används redan i stor utsträckning inom befintlig vård, de är väl etablerade nationellt och internationellt och innebär inga påtagliga risker för deltagarna. Valet att utföra träningen i personens hemmiljö, kan upplevas som ett intrång i den privata sfären, men det anses ha ringa etisk betydelse. Betydelsen av att träna i hemmet samt att forskningspersoner inte behöver ta sig till en mottagning anses överväga eventuell upplevelse av intrång i den privata sfären. Fysisk träning för sköra äldre skulle kunna utgöra en risk för den enskilde, studier på senare år visar dock att risken för detta är liten. Riskerna med fysisk inaktivitet bedöms vara lika stora eller större. Nära samarbete med ansvariga läkare på medverkande kliniker minskar ytterligare risken

för detta. Riskerna med fysisk aktivitet/träning anses inte heller vara större än de risker som patienten utsätts för i samband med operation. Styrketräning kan leda till träningsvärk, men deltagarna kommer att informeras om detta och att träningsvärk är en indikation på ökat muskelarbete vilket är en förutsättning för ökad muskelstyrka. Vid varje träningstillfälle kommer forskningspersonerna tillfrågas om eventuella komplikationer av träningsprogrammen, om sådana anges kommer intensiteten i träningen anpassas därefter. Frågorna om livskvalitet (EORTC QLQ-C30) är använda i en mängd studier, både i Sverige och internationellt och man har testat om de upplevs som oroväckande eller stötande, vilket inte varit fallet. Vi bedömer därför att patienterna i denna studie inte heller kommer att bli oroliga eller upprörda av frågorna.

5:2 Redogör för möjlig nytta för de forskningspersoner som ingår i projektet (gäller särskilt behandlingsforskning)

Deltagarna i studien ges möjlighet att få en strukturerad uppföljning av hälso- och funktionstillståndet vilket inte utförs i klinisk praxis och kan anses vara till fördel för forskningspersonen eftersom det ger dem en tydligare bild av deras fysiska funktionstillstånd. De som slumpas till träningsgruppen ges också möjlighet att förbättra sin fysiska funktion vilket kan ha betydelse för deras dagliga aktiviteter och deras tillfrisknande postoperativt.

5:3 Identifiera och precisera om eventuella etiska problem (fördelar/nackdelar) kan uppstå i ett vidare perspektiv genom projektet (Se p. 5:3 i Vägledning till ansökan)

Här kan redovisas om exempelvis vissa grupper (andra än de forskningspersoner som ingår i forskningsprojektet) kan komma att utpekas/få hjälp som ett resultat av studien.

Om studien visar på positiva effekter för deltagarna kan detta i ett vidare perspektiv även komma andra äldre personer till gagn när/om de kommer behöva genomgå buk- eller thoraxkirurgi. Etiska problem i former av risker i ett vidare perspektiv anses inte föreligga. Även om studien inte kan påvisa några positiva träningseffekter så är fördelarna med att öka den fysiska aktiviteten enbart postitivt med avseende på olika hälsofaktorer.

6. Redovisning av resultaten

6:1 Hur garanteras forskningshuvudmannen och medverkande forskare tillgång till data (anges vid t.ex. uppdragsforskning) och vem ansvarar för databearbetning och rapportskrivning?

(Se p. 6:1 i Vägledning till ansökan)

Endast huvudansvarig och övriga forskare angivna i ansökan kommer att ha full tillgång till data. Utöver doktorander och postdocs involverade i studien kommer medarbetare på medverkande kliniker kunna få tillgång till vissa data i samband med magisterarbeten, ST-uppsatser, då under handledning av huvud- och/eller medsökande.

6:2 Hur kommer resultaten att göras offentligt tillgängliga? Kommer studien att insändas för publicering i tidskrift eller publiceras på annat sätt? (Se p. 6:2 i Vägledning till ansökan)

Ange i vilken form resultaten planeras offentliggöras samt tidsplan för detta.

Resultaten kommer att publiceras i avhandlingar, vetenskapliga tidskrifter och i vetenskapliga uppsatser på avancerad nivå tex master, ST-uppsats etc, samt i populärvetenskapliga sammanhang riktat till vårdpersonal och allmänhet.

6:3 På vilket sätt garanteras forskningspersonernas rätt till integritet när materialet offentliggörs/publiceras?

Redovisas resultat på statistisk gruppnivå? Beskriv procedurer eller metoder för avidentifiering/anonymisering.

I samband med insamling av data avidentifieras alla deltagare, resultat kommer endast att presenteras på gruppnivå.

7. Redovisning av ekonomiska förhållanden och beroendeförhållanden

Redovisning enligt punkterna 7:1-7:3 syftar till att tydliggöra alla direkta eller indirekta förhållanden, som kan tänkas påverka forskarens relation till forskningspersonerna (vid t.ex. informations-, samtyckes-, genomförandeprocedurer).

7:1 Vid uppdragsforskning

Ange uppdragsgivaren t.ex. ett företag (vid klinisk läkemedelsprövning eller prövning av andra nya produkter), en organisation eller en myndighet.

Namn: Kontaktperson:

Adress: Telefon/mobiltelefon:

Ange uppdragsgivarens relation till forskningshuvudmannen/medverkande forskare, t.ex. anställningsförhållande

7:2 Redovisa eventuella ekonomiska överenskommelser med uppdragsgivare eller andra finansiärer (namn, belopp)

Vid klinisk läkemedelsprövning bör hänvisning ske till ingånget avtal med sjukvårdshuvudmannen. Liknande överenskommelser kan förekomma vid annan uppdragsforskning och bör redovisas på samma sätt. Separata överenskommelser med den/de som ska genomföra forskningen ska redovisas. Belopp som kommer att erhållas för studien/ersättning till kliniken/genomföraren, vad ersättningen bör täcka och ev. belopp som erhålls per forskningsperson, bör också anges här.

NA

7:3 Redovisa forskningshuvudmannens, huvudansvarig forskares och medverkande forskares egna intressen

Här redovisas t.ex. aktieinnehav, anställning, konsultuppdrag i finansierande företag, eget företag som kan få (direkt eller indirekt) ekonomisk vinst av forskningen.

NA

8. Undertecknande

Behörig företrädare för sökande forskningshuvudman enligt p. 1:2.

Ort: Huddinge Datum: 20150623

Signatur: [signature]

Namnförtydligande:

Tjänstetitel:

Maria Eriksdotter
Prefekt
Institutionen för Neurobiologi, vårdvetenskap och samhälle

Undertecknad forskare som genomför projektet (kontaktperson) enligt p. 1:3 intygar härmed att forskningen kommer att genomföras i enlighet med ansökan.

Ort: Huddinge Datum: 20150623

Signatur: Elisabeth [signature]

Namnförtydligande:

Tjänstetitel:

Regionala etikprövnings-
nämnden i Stockholm
Inkom: 2016 -08- 18
Dnr: 2016/1587-32

Komplettering till tidigare godkänd etisk ansökan Dnr 2015/1179-31.

Kompletteringen består av fyra delar: 1. Validering av instrumentet Patientspecifik funktionskala; 2. Förändring av tidigare angivet träningsprogram för att följa nya rön inom forskningsområdet; 3. En kompletterande intensifierad nutritionsintervention; 4. Intervjustudie.

1.
Instrumenten Patientspecifik funktionskala är översatt till svenska och testat på en svensk patientgrupp efter handkirurgi. Syftet med denna studie är att utvärdera instrumentets innehålls- och samstämmighetsvaliditet hos personer över 70 år som ska genomgå bukkirurgi. Studien kommer att genomföras inom ramen för en feasibility-studie som genomförs innan nedan beskriven och tidigare godkänd interventionsstudie startar. Forskningspersonerna fyller i formuläret tillsammans med ansvarig fysioterapeut vid första och sista besöket (se nedan). Övriga mätmetoder som ska användas i studien är angivna i tidigare godkänd ansökan.

2.
I tidigare godkänd forskningsplan angavs följande:

a) *"Deltagarna kommer att träna inspiratorisk muskelstyrka där de får andas mot motstånd och börja med 30% av deras maximala kapacitet. Träningen ska skattas som ansträngande (5 på Borgs CR-skala) och motståndet ökas/minskas i relation till detta. Träningen kommer att ske 2-3 ggr/vecka under 15 min."*

Då nya rön indikerar att träningen bör vara mer högintensiv men med kortare intervall kommer deltagarna nu att träna på 50-70% av deras maximala kapacitet och utföra 30 andetag x2 på denna nivå och skattas till 5-7 på Borgs CR-skala. Träningen kommer att ske dagligen, men under handledning av fysioterapeut 2-3 dagar/v.

b) *"Individanpassad funktionell träning.... Detta kommer att kombineras med funktionella övningar utifrån från varje individs behov som t.ex. att bära och förflytta saker."*

Detta kommer att kompletteras med att deltagarna tillsammans med fysioterapeut fyller i formuläret "Patientspecifik funktionskala" där deltagaren får beskriva tre viktiga aktiviteter som de har problem med att utföra eller inte kan utföra alls. De får sedan skatta hur svårt de tycker det är att utföra aktiviteten på en skala från 0=kan inte utföra aktiviteten till 10=kan utföra aktiviteten obehindrat. Dessa aktiviteter kommer sedan att införlivas i träningsprogramet och följas upp efter avslutad intervention. Se bilaga 1 för uppdaterad patientinformation.

3.
Syfte
Att utvärdera om en kombination av preoperativ fysisk aktivitet och en intensifierad nutritionsbehandling inför buk-kirurgi ökar aptit och näringsintag och därmed förhindrar nedbrytning av muskelmassa och funktion hos äldre patienter med risk för undernäring.

Områdesöversikt
Antalet sköra äldre ökar i takt med en åldrande befolkning. Proteinbehovet ökar med stigande ålder, medan sjukdom och behandling innebär flera försvårande faktorer för aptit och näringsintag. Undernäring är därför ett uttalat problem före och efter avslutad behandling. Förlust av muskelmassa och funktion kan vara direkt skadligt och kan associeras med sämre

prognos. Styrketräning kan förbättra muskelfunktion och balans hos äldre sköra personer, men kan å andra sidan försätta en patient i ett katabolt tillstånd om inte energi och protein tillförs i tillräcklig mängd. Datortomografi, DT används generellt för att identifiera förekomst av tumör men ej i utredning av undernäring.

Metod
Design; Interventionsstudie
Datakälla; Studien är en randomiserad kontrollerad studie där deltagarna randomiseras till intervention bestående av preoperativ träning eller kontrollgrupp bestående av sedvanlig behandling. **Tillägg:** Patienter som screenas för risk för undernäring i båda studiearmarna kommer att erbjudas en intensifierad nutritionsbehanding av dietist. Enligt powerberäkning för effekter på nutrionsvariabler krävs 40 patienter/arm i RCT studien.
Metod; Screening av risk för undernäring sker med hjälp av tre frågor enligt rekommendation från SKL: ofrivillig viktförlust; ätsvårigheter; och BMI.
De patienter som befaras ha en risk för undernäring ombedes fylla i en tre dagars kostdagbok och bokas in för dietistkontakt vid; **1)** nybesök **2)** vid tid för operation **3)** 8 veckor efter hemgång, och **4)** 1 år efter operation. Därtill telefonuppföljning/stöd varje vecka fram till operation. Intensifierad nutritionsbehanding av dietist innebär en utredning av kostintag och ev ätproblem och inkluderar individuellt anpassade kostförslag med fokus på energi- och proteinrik mat och dryck samt måltidsordning.
Vid behov förskrivs proteinrika kosttillägg som alla är upphandlade och godkända för SLL. För att utvärdera energi och proteinintag kommer kostdataprogram DietistNet att användas. Detta ger oss samtidigt en möjlighet attt studera följsamhet till given kostbehandling samt mängd konsumerade kosttillägg.
Befintliga DT-bilder ska analyseras av dietist för att fastställa kroppssammansättning och ev förändringar av denna, vid nybesök och ett år efteråt. DT-bilderna tas redan i reguljär vård och kräver ingen extra undersökning av patienterna. Se bilaga 1 för uppdaterad patientinformation.

Betydelse: Få studier har undersökt hur kost och näringsintag kan påverkas av fysisk aktivitet hos sköra äldre, trots att vi vet att undernäring är ett problem i sjukvården. Följsamhet till ordinerad kostbehandling och kosttillägg är sällan studerat. DT tas för att fastställa diagnos och för att följa och utvärdera behandling. Med resultatet av mätningarna kan vi fastställa graden av muskelförlust och observera specifika vävnadsförändringar utan extra besvär för patient eller kostnad för sjukvården.
Om detta tillägg i studien visar en positiv effekt, kan denna tilläggsansökan leda till ett ökat samarbete mellan dietister och fysioterapeuter i såväl öppen- som slutenvård med en stärkt vårdkedja, förbättrat omhändertagande och framförallt förbättrad vård för äldre sköra patienter som genomgår avancerad kirurgi.

Etiska överväganden: Originalstudien är godkänd enligt regionala etikprövningsnämnden i Stockholm (Dnr 2015/1179-31) men en tilläggsansökan behövs för den specifika nutritionsinterventionen. Studiepatienter kommer i enlighet med originalstudien erhålla muntlig och skriftlig information och informerat samtycke kommer inhämtas innan inkludering. Valda metoder och mätinstrument är validerade och används i liknande studier på andra patientgrupper.

4.
Områdesöversikt
Tidigare studier indikerar att optimering av fysisk funktion och nutritionstillstånd innan operation kan minska risken för komplikationer. Patienter vill gärna få sin operation genomförd så snart som möjligt och de standardiserade vårdförloppen som nu införs i Sverige inom cancervården medför att tid för optimering blir kort. Det finns dock inga medicinska indikationer som tyder på att en förskjutning ytterligare några veckor medför några risker för patienten i form av sämre förväntat utfall i cancersjukdomen.

Syfte
Studien syftar till att beskriva patienters syn på att skjuta på operationen ett par veckor för att kunna träna upp sin fysiska funktion och förbättra sitt nutritionstillstånd för att eventuellt minska risken för komplikationer och nedsatt funktion.

Metod
Ca 20-30 forskningspersoner som uppfyller kriterierna för inklusion i den pågående observations-studien kommer att tillfrågas om deltagande samtidigt som de tillfrågas om deltagande i observationsstudien (Dnr 2015/1179-31). Muntlig och skriftlig information lämnas ut av forskningssjuksköterska och skriftligt samtycke inhämtas av ansvarig fysioterapeut (se bilaga 2). Både de som tackar ja och nej till observations-studien kommer inkluderas. Intervjuerna kommer att ske innan operation. Samtliga intervjuer spelas in med en diktafon. Inspelade intervjuer transkiberas och analyseras med lämplig metod. En kodlista upprättas över deltagande personer och listan förvaras inlåst och på annat ställe än ljudinspelningarna och transkripten. Endast studieledningen har tillgång till kodlistan.

Etiska överväganden
Det är viktigt att framhålla att forskningspersonernas deltagande i studien är frivilligt och att de när som helst kan avbryta sin medverkan i och under intervjun. Intervjufrågorna skulle kunna upplevas som ett intrång i den privata sfären, men detta kan vägas mot att forskningspersonen får möjlighet att uttrycka sig och beskriva sin situation genom att delta i projektet. Data presenteras utan att enskilda personer kan identifieras.

Huddinge 160810

Elisabeth Rydwik, forskningsansvarig
Inst. NVS Karolinska Institutet

Ann Langius-Eklöf
~~Maria Eriksdotter~~, prefekt St-f
Inst. NVS, Karolinska Institutet

GODKÄNNES Dat. 2016 -08- 2 6

Hans Glaumann
Vetenskaplig sekreterare
Regionala etikprövningsnämnden
i Stockholm

BESLUT

Dnr: 2015/1179-31
Sökande: Karolinska Institutet
Behörig företrädare: Maria Eriksdotter
Projekt: Better in Better out: Preoperativ träning för att minska risken för komplikationer och ge kortare vårdtider för att sköra äldre efter buk-och thoraxoperation
Forskare som genomför projektet: Elisabeth Rydwik

Nämnden har vid sammanträdet den 19 augusti 2015 lämnat över till den vetenskaplige sekreteraren att avgöra ärendet sedan kompletteringar gjorts.

Sedan sökanden kommit in med begärda kompletteringar fattar den vetenskaplige sekreteraren följande

BESLUT 2015-09-02

Nämnden godkänner forskningen.

På nämndens vägnar

Hans Glaumann
vetenskaplig sekreterare

Beslut expedierat till behörig företrädare
Kopia för kännedom till ansvarig forskare

Postadress FE 289 171 77 STOCKHOLM | **Besöksadress** Tomtebodavägen 18A Solna | **Telefon** 08-524 870 00 (vx) | **Fax** 08-524 866 99 | **E-post** kansli@stockholm.epn.se | **Hemsida** www.epn.se
